# Supplementary material for: Integrating AlphaFold2 models and clinical data to improve the assessment of Short Linear Motifs (SLiMs) and their variants’ pathogenicity
Source: PLoS Comput Biol. 2025 Aug 4;21(8):e1012829. doi: 10.1371/journal.pcbi.1012829 (PMC12338786; doi:10.1371/journal.pcbi.1012829)
Supplement: S1 Text — Figure A SI. Flowchart of the MotSASi pipeline, integrating crystallographic structures from the PDB and predictive models ftrom AlphaFold2. CSC = Clinical Significance Cycle (x = 1), SAC = Structural Analysis Cycle (x = 2), FPC = Flexible Position Cycle (x = 3), JSD = Jensen-Shannon Divergence, P = Positive set, N = Negative set, R = Remaining set. The CSC, SAC, and FPC cycles occur sequentially. Candidates in the S0 and Rx sets are assessed using ClinVar and gnomAD matrices in all three cycles, whereas candidates in the R1 and R2 sets are evaluated against the FoldX matrix exclusively in the SAC and FPC cycles. Figure B SI. Plots of functions and their corresponding formulas for (A) gnomAD, (B) PDB-derived, and (C) AlphaFold2 (AF2)-derived Confidence Scores. Figure C SI. (a) Histogram displaying the ClinVar and gnomAD variants used to define the ΔΔG stability threshold (in kcal/mol). Pathogenic variants are indicated in red, and benign variants in green. (b) ROC curve (AUC = 0.929) generated during the ΔΔG threshold determination process for PDB crystallographic structures. The red dot marks the threshold value of 2.1 kcal/mol, corresponding to a sensitivity of 83% and a specificity of 84%. Figure D SI. (a) Histogram of ΔΔG stability values (in kcal/mol) calculated on AlphaFold2 (AF2)-generated models for the same ClinVar and gnomAD variants previously classified based on the 2.1 kcal/mol PDB threshold. Non-tolerated variants are shown in red, tolerated variants in green. (b) ROC curve (AUC = 0.865) generated during the process of determining the ΔΔG cutoff value for AF2. The red dot marks the cutoff value of 1.6 kcal/mol, corresponding to a sensitivity of 81% and a specificity of 80%. Figure E SI. Comparison of structural (stability ΔΔG) SAS matrices obtained using either the crystallographic structure deposited in the Protein Data Bank (PDB) (upper panel) or the SLiM-receptor structure modeled by AlphaFold2 (AF2) (lower panel) for the LIG_PDZ_Class_1 ELM motif c [file pcbi.1012829.s001.docx]

**Supplementary Information**


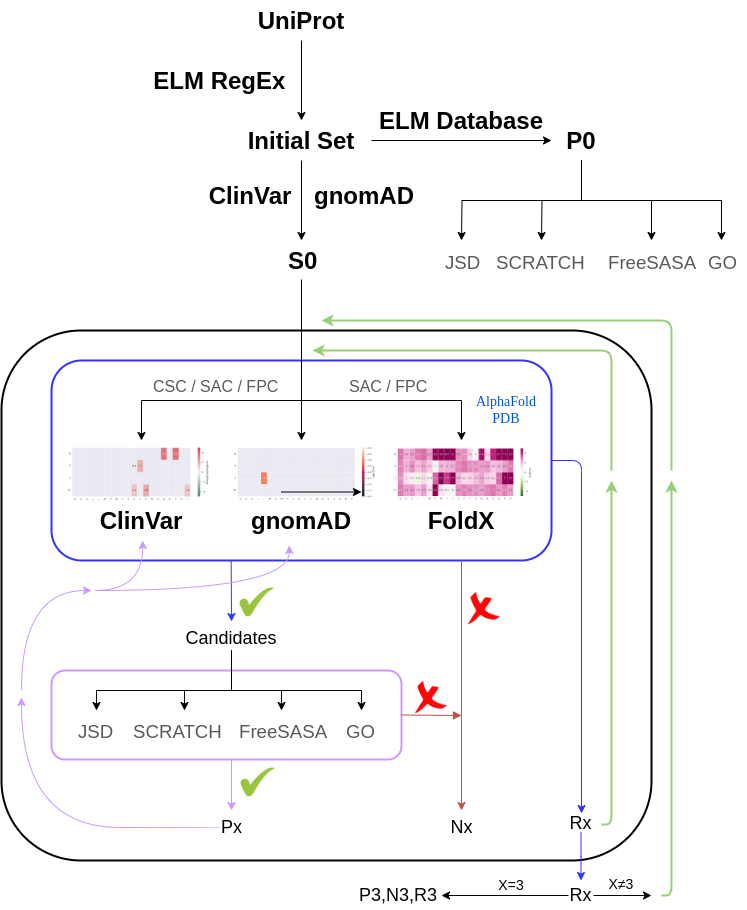


**Fig A:** Flowchart of the MotSASi pipeline, integrating crystallographic structures from the PDB and predictive models from AlphaFold2. CSC = Clinical Significance Cycle (x=1), SAC = Structural Analysis Cycle (x=2), FPC = Flexible Position Cycle (x=3), JSD = Jensen-Shannon Divergence, P = Positive set, N = Negative set, R = Remaining set. The CSC, SAC, and FPC cycles occur sequentially. Candidates in the S0 and Rx sets are assessed using ClinVar and gnomAD matrices in all three cycles, whereas candidates in the R1 and R2 sets are evaluated against the FoldX matrix exclusively in the SAC and FPC cycles.


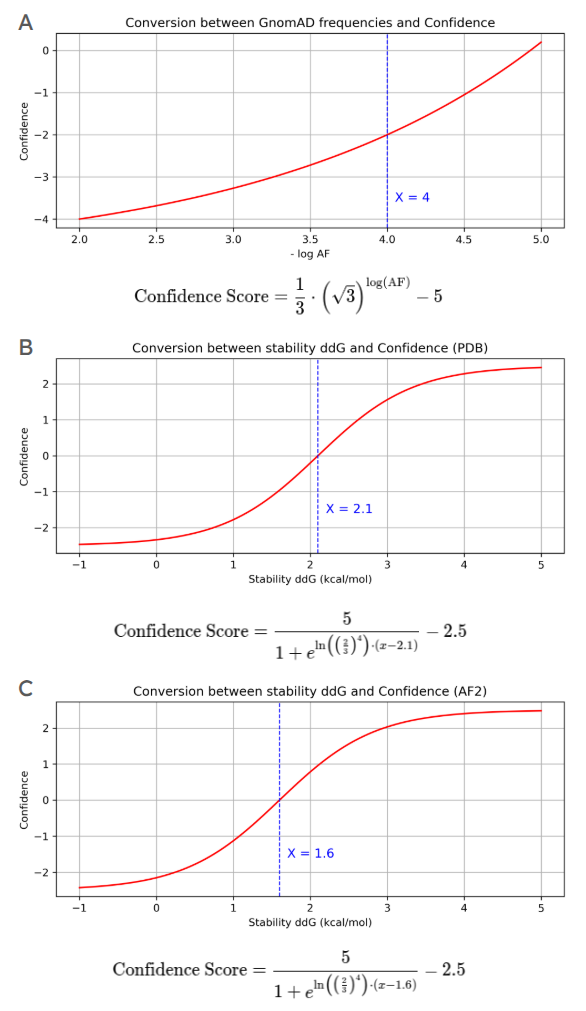


**Fig B:** Plots of functions and their corresponding formulas for (A) gnomAD, (B) PDB-derived, and (C) AF2-derived Confidence Scores.


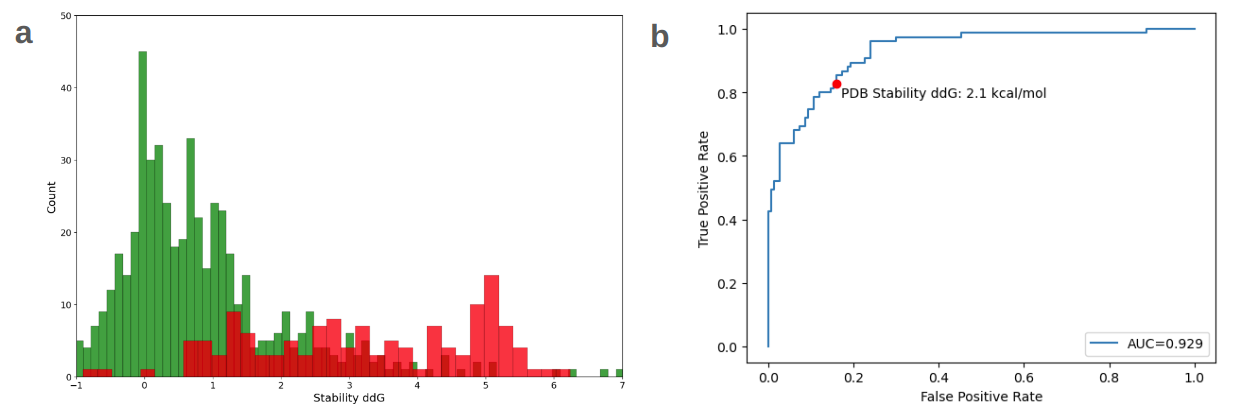


**Fig C:** (a) Histogram displaying the ClinVar and gnomAD variants used to define the △△G stability threshold in (kcal/mol). Pathogenic variants are indicated in red, while benign variants are shown in green. (b) ROC curve (AUC = 0.929) generated during the △△G threshold determination process for PDB crystallographic structures. The red dot marks the threshold value of 2.1 kcal/mol, corresponding to a sensitivity of 83% and a specificity of 84%.


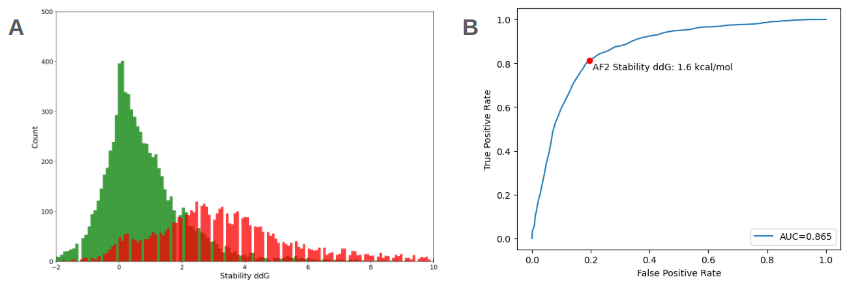


**Fig D:** (a) Histogram of △△G stability values (in kcal/mol) calculated on models generated by AF2 for the same ClinVar and gnomAD variants previously classified as tolerated or non-tolerated in PDB-deposited crystallographic structures, based on the previously mentioned **2.1 kcal/mol** threshold. Non-tolerated variants are shown in red, while tolerated variants are shown in green. (b) ROC curve (AUC = 0.865) generated during the process of determining the △△G cutoff value for AF2. The red dot marks the cutoff value of 1.6 kcal/mol, corresponding to a sensitivity of 81% and a specificity of 80%.


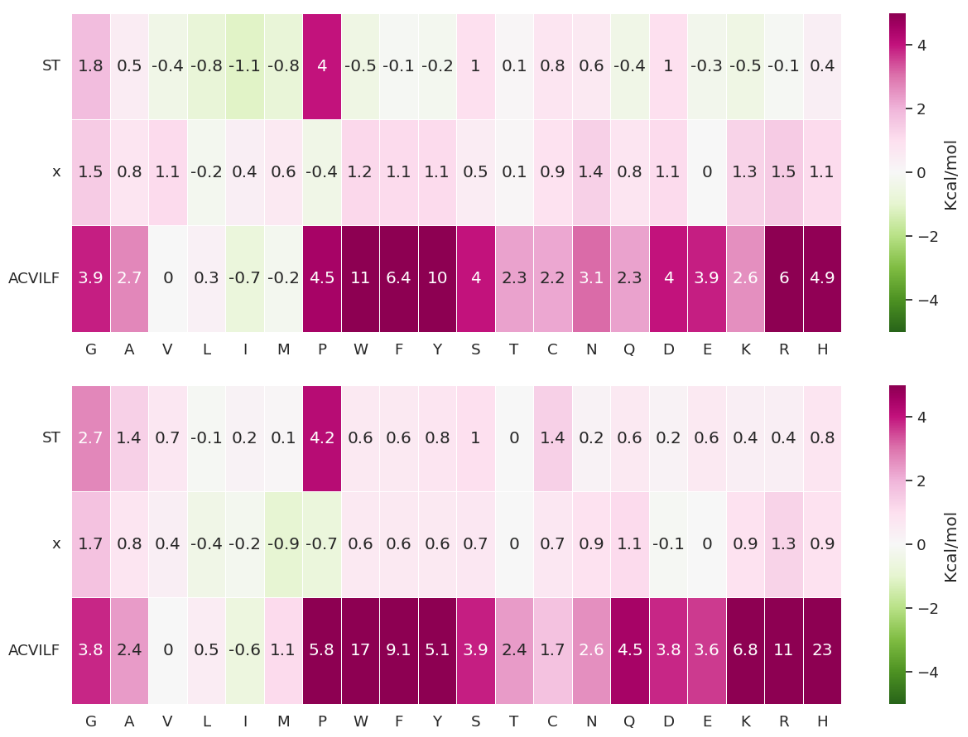


**Fig E:** Comparison of structural (stability ΔΔG) SAS matrices obtained using either the crystallographic structure deposited in the Protein Data Bank (PDB) (upper panel) or the SLiM-receptor structure modeled by AlphaFold2 (AF2) (lower panel) for the LIG_PDZ_Class_1 ELM motif class (regular expression: [ST].[ACVILF]$).


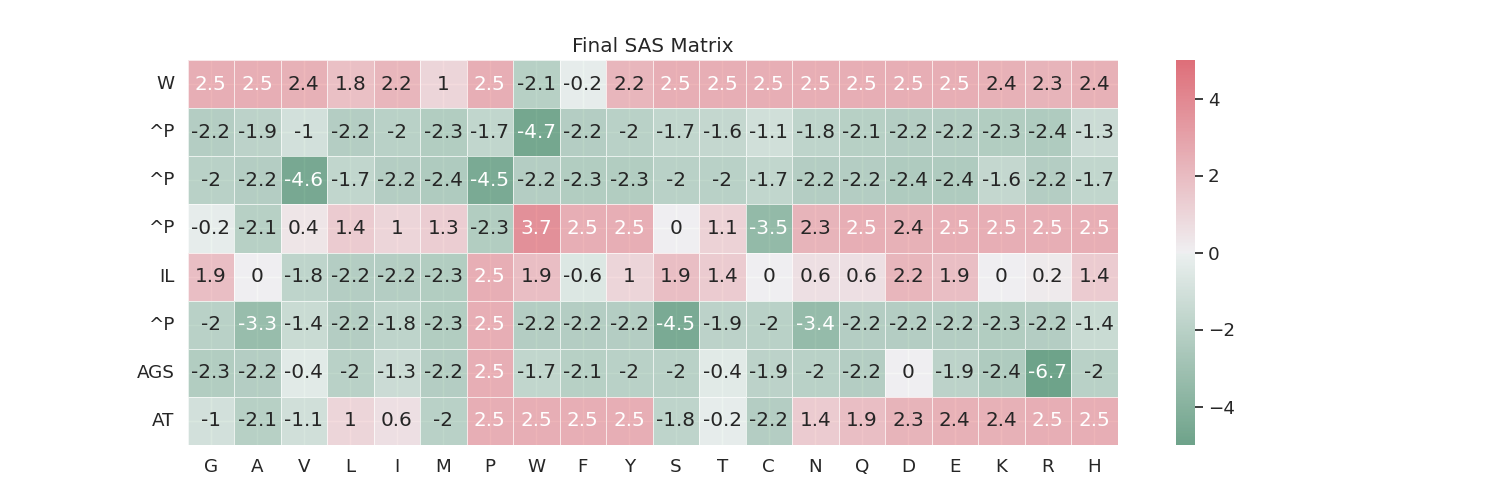


**Fig F:** Final SAS matrix with confidence scores in the cells for the LIG_CaM_NSCaTE_8 motif class (regular expression: W[^P][^P][^P][IL][^P][AGS][AT]).


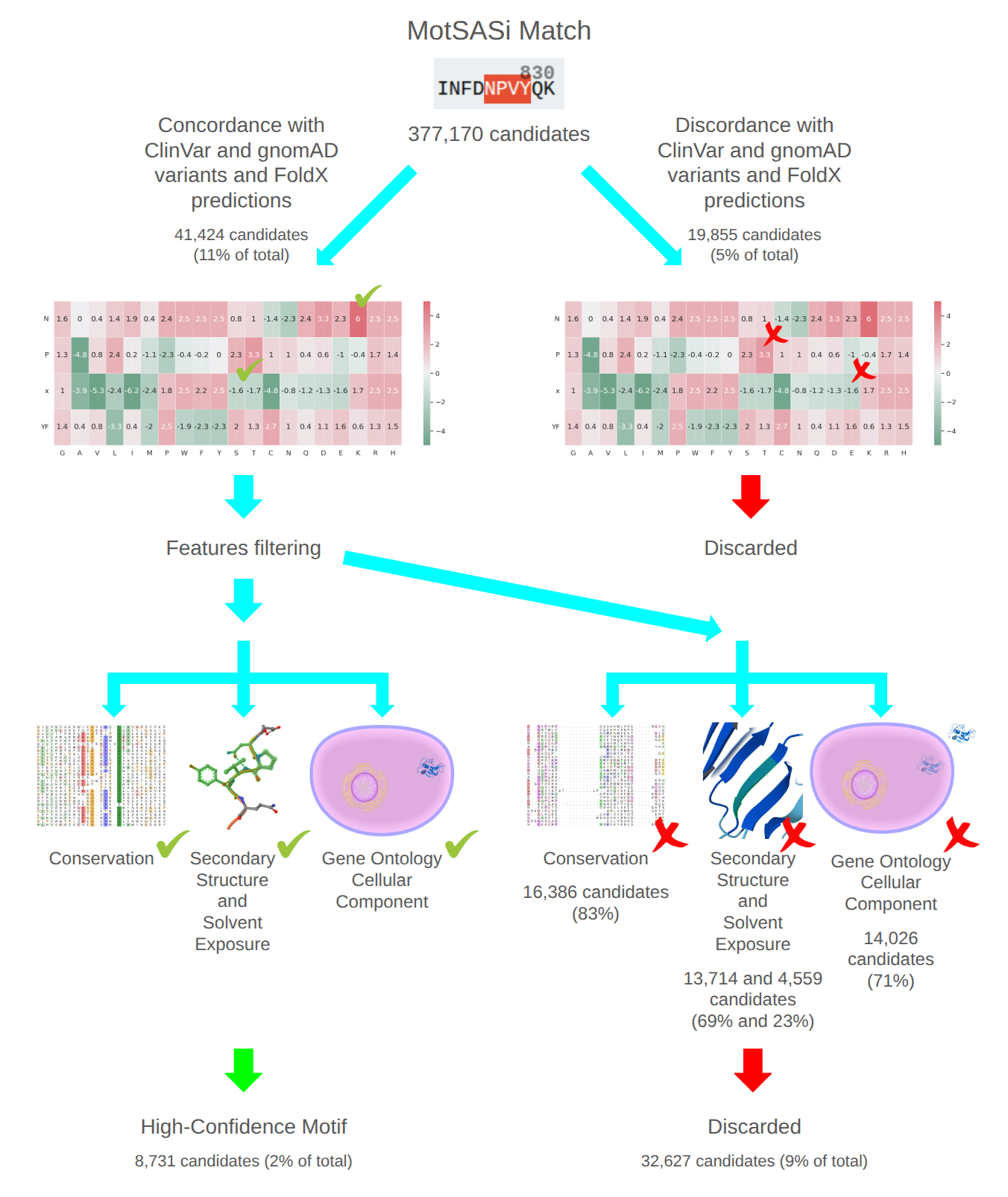


**Fig G:** Pipeline illustrating the filtering process applied by MotSASi. Values represent the aggregate data across all motif classes and instances evaluated.
