# Supplementary material for: Integrating AlphaFold2 models and clinical data to improve the assessment of Short Linear Motifs (SLiMs) and their variants’ pathogenicity
Source: PLoS Comput Biol. 2025 Aug 4;21(8):e1012829. doi: 10.1371/journal.pcbi.1012829 (PMC12338786; doi:10.1371/journal.pcbi.1012829)
Supplement: Attachment — Submitted filename: renamed_45276.pdf [file pcbi.1012829.s002.pdf]

Summary

The manuscript presents a computational workflow integrating AlphaFold2-derived structural models with clinical variant data to enhance the identification and pathogenicity assessment of Short Linear Motifs (SLiMs). The study builds upon previous work, introducing an improved version of MotSASi, which provides a comprehensive resource of SLiM-associated variants.

The study is highly relevant to computational biology and clinical genomics, particularly in interpreting variants of uncertain significance. The integration of AlphaFold2-generated structures addresses limitations in available crystallographic data and improves variant classification in SLiMs. However, several aspects of the manuscript could be clarified, justified, and refined to ensure accuracy, transparency, and reproducibility.

Major concerns and key areas for improvement

1. AlphaFold2 vs. AlphaFold3 – Modeling accuracy and reliability

- **Key concern:** The study is based on AlphaFold2 (AF2) and compares the MotSASi tool to AF2. AlphaFold3 (AF3) appeared in 2024, showing an enhanced multimeric modelling and improved interaction predictions, which could refine SLiM-receptor modelling beyond AF2's capabilities
    - AF3 is performing better in multimeric modeling, especially for transient interactions like SLiM-mediated protein interactions.
    - AF3 has more accurate binding site predictions, reducing false positives in SLiM-receptor modeling.
- **Recommendation:**
    - Authors should justify why AF2 was used instead of AF3.
    - If AF3 is unavailable for the MotSASi pipeline, discuss how its advancements could impact future studies.

2. Accessibility and usability of MotSASi

- **Key Concern:** The pipeline is available through a Zenodo link, but the manuscript does not provide clear instructions on how users can access or implement MotSASi. Providing a public GitHub repository with all scripts and comprehensive documentation would significantly enhance its usability.
- **Recommendation:**
    - Provide details on how to run AF2-based modeling in a reproducible way.

Further concerns and suggestions

Introduction

- If it is possible, explicitly mention the false-positive rate in common SLiM prediction tools
- Instead of providing a detailed description of the researchers' previous publication on MotSASi, summarize its key findings in a single paragraph. Also, include information on its adoption, such as its impact on motif research.
- The introduction should end with a clear hypothesis statement.

Methods

- Some sections are very short, including only 1-2 sentences (eg. Secondary structure prediction, Residues Exposure calculation, Gene Ontology terms, Protein structure and matrices visualisation)
- Threshold justification for pLDDT (>65) and interaction energy (< -5 kcal/mol) thresholds is not well-described. Were these values empirically determined in a preliminary study?
- Specify which ELM database version was used.
- Running AF2 is computationally expensive. Provide details on:
    - Runtime estimates and hardware specifications
    - Alternative precomputed structure sources
- Consider adding a diagram illustrating the iterative filtering process.

Results

- Functional significance of newly identified SLiMs should be discussed.
- MotSASi vs. AlphaMissense comparison raises several questions
    - Was AlphaMissense evaluated on a dataset that inherently favors MotSASi?
    - AlphaMissense is trained on a broad dataset including variants across all protein regions, not just those within SLiMs. As MotSASi is designed specifically for these regions, it may be optimised for this niche. If AlphaMissense were retrained using only SLiM-associated variants, would it learn better patterns specific to SLiMs?
    - MotSASi misses more pathogenic variants than AlphaMissense (49 vs. 27), raising potential clinical limitations.

Discussion

- The tool's limitations are poorly discussed.
    - The paper should acknowledge that AF2 has challenges in modeling transient interactions and disordered regions.
    - The authors should discuss whether AlphaFold3 could further improve SLiM modeling.
    - Alternative structure prediction tools (e.g., RoseTTAFold, ESMFold) could be mentioned as future directions.
- Statements about MotSASi outperforming AlphaMissense should be more nuanced.

- Instead of conclusive superiority, highlight specific strengths and areas where both tools can improve.
