# Supplementary material for: Integrating AlphaFold2 models and clinical data to improve the assessment of Short Linear Motifs (SLiMs) and their variants’ pathogenicity
Source: PLoS Comput Biol. 2025 Aug 4;21(8):e1012829. doi: 10.1371/journal.pcbi.1012829 (PMC12338786; doi:10.1371/journal.pcbi.1012829)
Supplement: Attachment — Submitted filename: MotSASi2.0_Response_to_Reviewers.pdf [file pcbi.1012829.s004.pdf]

Buenos Aires, April 10, 2025

Ref Ms# PCOMPBIOL-D-25-00157

Dear Dr. Mohammad Sadegh Taghizadeh, Ph.D., Academic Editor, & Dr. Ilya Ioshikhes, Section Editor
PLOS Computational Biology

We are pleased to submit the revised version of our manuscript, titled “Integrating AlphaFold2 Models and Clinical Data to Improve the Assessment of Short Linear Motifs (SLiMs) and Their Variants’ Pathogenicity” by Brunello et al.

We have carefully reviewed and addressed all comments and suggestions provided by the reviewers, which have been invaluable in enhancing the quality and clarity of our work. Accordingly, we have revised and rewritten the manuscript as needed. A detailed, point-by-point response to the reviewers' comments, along with a summary of the changes—highlighted in red in the revised manuscript—is provided below.

We appreciate your time and consideration.

Best Regards,

Bioq. Franco Gino Brunello
Department of Biological Chemistry, Faculty of Exact and Natural Sciences, University of Buenos Aires (FCEyN-UBA), and Institute of Biological Chemistry (IQUIBICEN), National Scientific and Technical Research Council (CONICET)

Specific answers to the Reviewer #1

Reviewer #1: General Comment

The manuscript presents a computational workflow integrating AlphaFold2-derived structural models with clinical variant data to enhance the identification and pathogenicity assessment of Short Linear Motifs (SLiMs). The study builds upon previous work, introducing an improved version of MotSASi, which provides a comprehensive resource of SLiM-associated variants.

The study is highly relevant to computational biology and clinical genomics, particularly in interpreting variants of uncertain significance. The integration of AlphaFold2-generated structures addresses limitations in available crystallographic data and improves variant classification in SLiMs. However, several aspects of the manuscript could be clarified, justified, and refined to ensure accuracy, transparency, and reproducibility.

We sincerely appreciate the reviewer's positive assessment of our manuscript and his/her recognition of its relevance to computational biology and clinical genomics. We acknowledge the need for further clarification, justification, and refinement in certain aspects of the study, as pointed out by the reviewer. Accordingly, we have carefully revised and improved the manuscript to enhance its accuracy, transparency, and reproducibility. Below, we provide specific responses and explanations addressing each of the reviewer's, as well as other reviewers' suggestions.

Major concerns and key areas for improvement

1. AlphaFold2 vs. AlphaFold3 – Modeling accuracy and reliability

● Key concern: The study is based on AlphaFold2 (AF2) and compares the MotSASi tool to AF2. AlphaFold3 (AF3) appeared in 2024, showing an enhanced multimeric modelling and improved interaction predictions, which could refine SLiM-receptor modelling beyond AF2's capabilities

○ AF3 is performing better in multimeric modeling, especially for transient interactions like SLiM-mediated protein interactions.

○ AF3 has more accurate binding site predictions, reducing false positives in SLiM-receptor modeling.

● Recommendation:

○ Authors should justify why AF2 was used instead of AF3.

○ If AF3 is unavailable for the MotSASi pipeline, discuss how its advancements could impact future studies.

We agree with the reviewer that current information as revealed by the literature suggests that AF3 could be a more accurate tool (compared with AF2) for building the SLiM-receptor complexes. However, although AF3 was announced on May 2024, it could only be used for some tests on-line, and its parameters (weights and biases) became available only later (November 2024). At that moment most of our calculations were already done and we were in the process of writing the manuscript.

Also important is the fact that we made a lot of effort to enable the selection of the correct AF2 model, i.e. the one that properly positions SLiMs. And as our results show, we have much confidence that when the AF2 model surpasses both SLiM-receptor and pLDDT score herein defined thresholds, and also no contradictions are presented between structural and variant matrix, AF2 correctly builds the complex. Nonetheless, and in line with the reviewer's comment/suggestion, we have commented in the revised version of the manuscript in the discussion, about the potential impact of AF3 in the MotSASI pipeline.

Specifically, on page 28 (Lines 632-643) of the revised version, we included the following paragraph: "It is also important to mention that during the writing process of this work, AlphaFold3 (AF3) appeared in the public domain. Although initially (May 2024), it could only be used for limited online tasks, the full set of parameters was released in November 2024. AF3 is expected to outperform its predecessor in multimeric modeling and provide more accurate peptide-protein binding site predictions, potentially enhancing MotSASi's accuracy. On the other hand, incorporating AF3 into MotSASi's pipeline would serve as an additional test for its structural prediction capabilities, particularly in the context of comparing the structural and clinical substitution matrices. Moreover, it has been documented that AF2 still struggles to accurately predict the conformation of intrinsically disordered protein regions, where, as previously mentioned, several SLiMs occur [43]. These cases might also benefit from AF3."

2. Accessibility and usability of MotSASi

● Key Concern: The pipeline is available through a Zenodo link, but the manuscript does not provide clear instructions on how users can access or implement MotSASi. Providing a public GitHub repository with all scripts and comprehensive documentation would significantly enhance its usability.

● Recommendation:

○ Provide details on how to run AF2-based modeling in a reproducible way.

We fully agree with the reviewer's concern and appreciate the suggestion to enhance the accessibility and usability of MotSASi. In response, we have created a public GitHub repository containing all relevant Python scripts and comprehensive documentation to facilitate reproducibility and user implementation (the repository is available at https://github.com/fginob1/MotSASi2.0). This repository ensures that other researchers can easily run and test MotSASi, including the AlphaFold2-based modeling steps. Details on how to access and execute the pipeline have been included in the revised manuscript, in the Data Availability Statement (Page 32: Lines 728-731), which now states: " The data supporting the findings of this study, along with the custom Python 3 scripts, are available at: https://github.com/fginob1/MotSASi2.0 . DOI: 10.5281/zenodo.15189546"

Further concerns and suggestions

Introduction

● If it is possible, explicitly mention the false-positive rate in common SLiM prediction Tools

We appreciate the reviewer's suggestion and have included, in the revised manuscript, an explicit mention of the false-positive rate in common SLiM prediction tools. Based on our results, we estimate the false-positive rate to be approximately 90%. This aligns with general findings in the literature, where studies on similar problems report quite high false-positive rates, though no specific numbers are usually provided. Notably, our estimation is consistent with the value of 0.122 reported by Idrees et al. (DOI: 10.7717/peerj.5858/correction-1) for random expectation in Y2H experiments involving true domain-motif interactions. In direct relation to this point we added the following statement on page 4 (Lines: 88-94) of the revised version: "Our estimation, based on the results of our previous work, suggests that the false-positive rate could exceed 80% (5,056 high-confidence potential motifs containing clinically relevant variants, compared to 29,428 total sequence hits across the human proteome that also carry clinically relevant variants, identified using regular expressions for 20 motif classes). This estimate is consistent with the findings of Idrees et al., based on the ratio of true binders to non-binders in yeast two-hybrid systems using randomly generated peptides [13]."

● Instead of providing a detailed description of the researchers' previous publication on MotSASi, summarize its key findings in a single paragraph. Also, include information on its adoption, such as its impact on motif research.

According to the reviewer's suggestion we have, in the revised version of the manuscript, condensed (or shortened) the information of our previous publication on MotSASi. We also mentioned some information related to its adoption.

Specifically, the new version paragraph states (Page 5, Lines: 103-117):

"MotSASi leverages the structural information of the SLiM-receptor complexes to analyze the effects of single amino acid substitutions (SAS) within the motif, employing the FoldX software [15,16]. This analysis is further integrated with the clinical significance and population allele frequency of motif located missense variants [17,18]. The underlying premise is that non-functional SLiMs identified by chance through regular expression matching (false positives), exhibit a variation pattern—pathogenic (non-tolerant) versus benign (tolerant)—that significantly differs from that of functional SLiMs (true positives). The initial implementation yielded promising results, achieving significant enrichment of true positives, while also filtering out a substantial number of false positives. However, the algorithm was constrained to classifying only candidates with available crystallographic structures of motif-domain complexes, thereby limiting the analysis to just 10–20% of all motif matches in the proteome. MotSASi has been recognized as a valuable tool for characterizing intrinsically disordered regions, such as amyloid-beta, and has been well received by the research community [19,21]."

● The introduction should end with a clear hypothesis statement.

We agree with the reviewer on this point, and have corrected the manuscript accordingly. The revised version introduction now ends with the following statement explicitly, and clearly stating our hypothesis.

Page 6 (Lines 136-143): “In the present study, we hypothesized that AlphaFold's structural prediction capabilities would allow the generation of models for all human SLiMs, in complex with their domain-binding partners for those motif classes lacking representative crystallographic structures. Furthermore, we propose that integrating the resulting SAS structural tolerance matrix with the increasingly available allele frequency and clinical variant data would lead to: i) a significant increase in the coverage of potential true SLiMs in the human proteome, and ii) an improved method for predicting the pathogenicity of missense variants located within SLiMs.”

Methods

- Some sections are very short, including only 1-2 sentences (eg. Secondary structure prediction, Residues Exposure calculation, Gene Ontology terms, Protein structure and matrices visualisation)

In accordance with the reviewer’s comments, and in line with the suggestions made by the other reviewer, we have significantly expanded the methods section to provide further details for all mentioned technical steps.

For the specific mentioned point, the revised version of the method, presently reads (Pages 9-11, Lines 228-265):

Conservation Score calculation

Multiple sequence alignments (MSAs) were constructed using sequences from the UniRef50 cluster associated with each match-containing protein. The alignments were performed using MAFFT software and subsequently used to calculate the motif conservation score, determined through the Jensen-Shannon Divergence algorithm [29,30].

Secondary Structure prediction

Protein secondary structure prediction was performed using the Scratch software (an ensemble prediction method) with the full sequence provided as input [31]. The specific output utilized in this study was SSpro (release 6.0, 2021), which provides per-residue classification into three categories: helix, strand, or other. The implementation in Python 3 was carried out following the specifications provided in its official GitHub repository [32].

Residues Exposure calculation

The solvent-accessible surface area (SASA) was calculated using the FreeSASA library, whose implementation in Python 3 was carried out following the specifications indicated in its GitHub repository [33]. Calculations were performed on all AF2 provided models of the human proteome, available on its website. For each residue, the SASA was compared to that of a completely solvent exposed residue, and classified as either exposed or buried. The classification threshold was determined from each residue SASA probability distribution as derived from analysis of thousands of proteins. Thresholds for each of the 20 residues are available in the SASA folder of the MotSASi2.0 GitHub repository.

Gene Ontology terms

Gene Ontology (GO) terms were retrieved from The Gene Ontology Resource website [34]. The file used was go.obo (September 2023), which was parsed in Python 3 using the obonet library. Terms related to cellular localization were extracted for each studied protein.

Protein structure and matrices visualization

Protein structure visualizations and substitution matrices were generated using Visual Molecular Dynamics (VMD) software, PyMOL Molecular Graphics System and the Seaborn library [35,36]. Substitution matrices were created using Seaborn's heatmap method, with precomputed ClinVar, gnomAD, FoldX, or final numeric matrices in TSV format as input.

● Threshold justification for pLDDT (>65) and interaction energy (< -5 kcal/mol) thresholds is not well-described. Were these values empirically determined in a preliminary study?

The threshold values for pLDDT (>65) and interaction energy (< -5 kcal/mol) were determined within the context of the present study, based on the analysis of the dot plot shown in Figure 1B. We have clarified this point in the revised version of the manuscript, including the following paragraph (Page 14, Lines 333-342):

“To address this issue and avoid using motif-receptor complexes with incorrectly positioned motifs, we analyzed several parameters of the AF2 models, in order to assess their ability to discriminate between correct and incorrect SLiM positioning. These parameters include the interaction energy between the motif and domain chains (computed as described in FoldX Free-Energy Change calculations in the Methods section) and the confidence of motif residues estimated by AlphaFold’s pLDDT metric. As shown in Figure 1B (right panel), correct models exhibit a higher mean pLDDT for AF2-predicted SLiM residues (82.8 ± 10.0 vs. 40.9 ± 14.0, $p < 1 \times 10^{-39}$) and a lower interaction energy between the receptor and the SLiM (-12.1 ± 4.1 kcal/mol vs. -3.1 ± 6.6 kcal/mol, $p < 1 \times 10^{-15}$).”

● Specify which ELM database version was used.

In the revised version we have specified which version of the ELM database was used. Specifically, we added the following sentence (on Page 7, Lines 156-159):

“Specific ELM databases utilized in this work include elm_instances.tsv, elm_classes.tsv, elm_interactions.tsv, and elm_interaction_domains.tsv (last accessed version corresponds to September 2024).”

● Running AF2 is computationally expensive. Provide details on:

■ Runtime estimates and hardware specifications

We have provided runtime estimates and hardware specifications in the revised version. We have clarified this point in the revised version of the manuscript, including the following paragraph (Page 8, Lines 194-199): “The runtime estimation for running AF2 depends on the sequence length. Our calculations show that processing a single residue takes 0.89 ± 0.14

seconds per residue for the first model and 0.83 ± 0.16 seconds per residue for the remaining four models. These estimations were obtained using the following hardware specifications: RYZEN 9 5900X processor (24 cores), 62 GB RAM, 4 TB storage, and an NVIDIA GeForce RTX 3050 GPU."

■ Alternative precomputed structure sources

Although it would be great to provide and analyze using alternative precomputed structure resources, to our knowledge, there are no resources with the required SLiM-receptor complexes. The most widely used resource, which is based on AF2, provides structures for all human proteins, but isolated and not in their complex with the corresponding SLiMs.

● Consider adding a diagram illustrating the iterative filtering process.

A diagram illustrating the iterative filtering process through the diverse cycles of the workflow has been added and is presented in Figure 1 SI (see Supplementary Information).
For a fast evaluation, the diagram is also presented below:

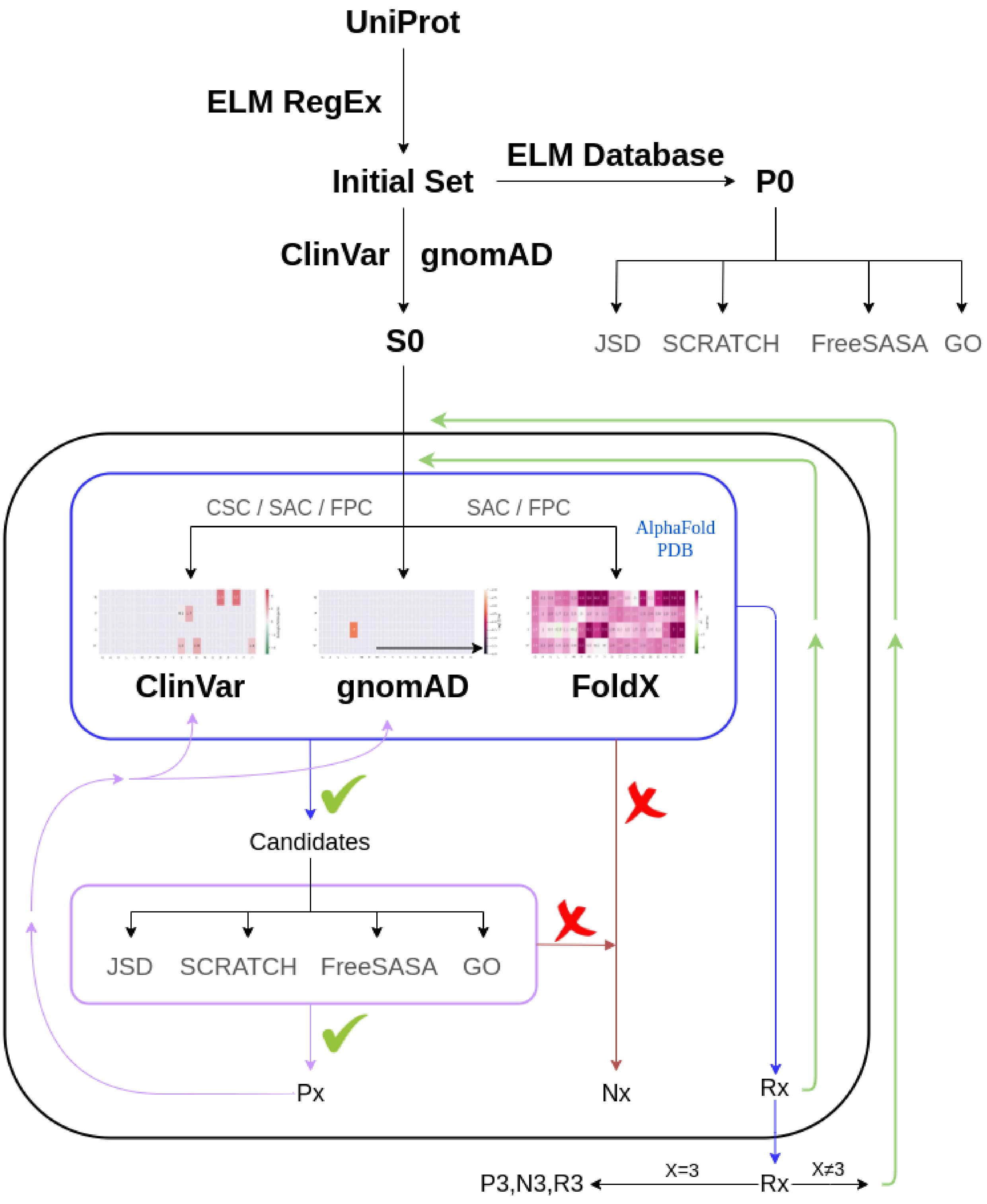

Figure 1 SI: Flowchart of the MotSASi pipeline, integrating crystallographic structures from the PDB and predictive models from AlphaFold2. CSC = Clinical Significance Cycle (x=1), SAC = Structural Analysis Cycle (x=2), FPC = Flexible Position Cycle (x=3), JSD = Jensen-Shannon Divergence, P = Positive set, N = Negative set, R = Remaining set. The CSC, SAC, and FPC

cycles occur sequentially. Candidates in the S0 and Rx sets are assessed using ClinVar and gnomAD matrices in all three cycles, whereas candidates in the R1 and R2 sets are evaluated against the FoldX matrix exclusively in the SAC and FPC cycles.

Results

- The functional significance of newly identified SLiMs should be discussed.

We agree with the reviewer that it would be interesting to discuss further the functional significance of newly identified SLiMs.

We added the following paragraph on Page 21 (Lines 466-472): "Nevertheless, as a result of our work, we now have access to a large number of newly identified motif instances or SLiMs, many of which are located in disease-associated OMIM genes, underscoring their potential functional significance. Their identification provides, therefore, a working hypothesis for further studies, seeking to confirm this function in the proper cellular and physiological context, while also providing a more detailed protein`s functional annotation (see below for the results concerning two particular examples)."

- MotSASi vs. AlphaMissense comparison raises several questions
  - Was AlphaMissense evaluated on a dataset that inherently favors MotSASi?

As stated in the methods section, the dataset used to compare MotSASI and AlphaMissense involved randomly setting aside a subset comprising 20% of ClinVar pathogenic and gnomAD benign variants found in ELM instances (true positives) and/or newly identified high-confidence motifs. Indeed, in the revised version, we have further and more clearly explained how the data set was built (both in the Methods section, Comparison Against AlphaMissense: Evaluation Pipeline Design, and again in the Results section, SLiM-receptor complex models improve SLiM variant pathogenicity prediction). Although we believe the set is unbiased, it is important to note, that since we are focusing on variants that ARE present in SLiMs, which, as we described, often occur in structurally disordered or loop regions, this could represent some intrinsic bias. In any case, and based on the reviewer's comment, we have added a comment on this issue in the discussion in the revised version of the manuscript.

Specifically, we added on Page 13 (Lines 308-316) the following paragraph:

"

Comparison Against AlphaMissense: Evaluation Pipeline Design

To perform a comparison with the bioinformatic pathogenicity predictor AlphaMissense, we developed a modified pipeline. In each cycle and iteration, a subset of 20% of ClinVar pathogenic variants and gnomAD benign variants, identified in ELM instances (true positives) or newly detected high-confidence motifs, is set aside to form an independent evaluation set. These variants are excluded from the pipeline and subsequently used to assess the performance of the MotSASi SAS matrices in comparison with AlphaMissense predictions, utilizing metrics such as accuracy, F1-score, and Matthews Correlation Coefficient (MCC).

"

And on Page 30 (Lines 695-698) we added: “Although the dataset built for this purpose was constructed in an unbiased manner, we must acknowledge that all tested variants were selected from SLiMs, which frequently reside in disordered or loop regions, where AM has been reported to show lower performance.”

○ AlphaMissense is trained on a broad dataset including variants across all protein regions, not just those within SLiMs. As MotSASi is designed specifically for these regions, it may be optimised for this niche. If AlphaMissense were retrained using only SLiM-associated variants, would it learn better patterns specific to SLiMs?

That’s a very interesting question and directly related to our previous answer. In our opinion, it could be possible that if AlphaMissense were retrained using only SLiM-associated variants, it could learn better the underlying patterns and improve its accuracy in predicting SLiM variant pathogenicity (even if it has been trained with data from individual protein models). Given the reviewer’s comments and valuable insights, we have decided to include a comment on this issue in the discussion section of the revised manuscript.

On Page 31 (Lines 705-707) we added “Given that AM has been trained on a vast dataset of missense variants, it is likely that its performance would improve if trained exclusively on SLiM-residing missense variants.”

○ MotSASi misses more pathogenic variants than AlphaMissense (49 vs. 27), raising potential clinical limitations.

We appreciate this comment and have addressed this concern in the manuscript. MotSASi adopts a more conservative approach compared to AlphaMissense, which aligns with ClinGen recommendations. While this may lead to missing some pathogenic variants, we believe this is advantageous in a clinical setting, where false negatives are generally preferable to false positives. Furthermore, we expand on this point in the discussion, emphasizing that in clinical genomics, it is more prudent to withhold reporting a variant until stronger evidence is available, rather than risk premature reporting and subsequent retraction. This aligns with best practices in molecular diagnostics, prioritizing reliability and minimizing potential misclassification risks.

On Page 24 (Lines 538-546) to the following statement: “Analysis of the confusion matrix shows that the key to the observed improvement in MotSASi lies in its ability to avoid tagging known benign variants as pathogenic. The naturally imbalanced dataset, with many more benign variants as often observed in real-world examples, results in AM misclassifying 607 benign variants as pathogenic, whereas MotSASi shows only 5 cases of these potential false positives. As expected, for known pathogenic variants, while MotSASi fails to detect 49, AM only fails in 27 cases. Generally speaking, it appears that MotSASi tends to be more conservative than AM concerning pathogenic classification, which aligns with current ClinGen recommendations.”, we add page 24 (Lines 546-549): “The overall data show that with MotSASi, we have developed an ideal tool for studying missense variants residing in functional SLiMs, even when a

crystallographic structure has not been deposited in the PDB, provided we can generate an optimal model with AF2."

And on Page 30 (Lines 695-705) "Although the dataset built for this purpose was constructed in an unbiased manner, we must acknowledge that all tested variants were selected from SLiMs, which frequently reside in disordered or loop regions, where AM has been reported to show lower performance. Analysis of the confusion matrices generated by each method revealed that, while AM provides more accurate predictions for pathogenic variants in the test set, it also produces many false positives. Beyond the "data problem," it is important to emphasize that in clinical genomics, false negatives are generally preferable to false positives. Extrapolated to molecular diagnosis, it is more prudent to withhold reporting a variant until stronger evidence is available, rather than report prematurely and risk subsequent retraction."

Discussion

● The tool's limitations are poorly discussed.

○ The paper should acknowledge that AF2 has challenges in modeling transient interactions and disordered regions.

○ The authors should discuss whether AlphaFold3 could further improve SLiM modeling.

○ Alternative structure prediction tools (e.g., RoseTTAFold, ESMFold) could be mentioned as future directions.

● Statements about MotSASi outperforming AlphaMissense should be more nuanced.

○ Instead of conclusive superiority, highlight specific strengths and areas where both tools can improve.

We agree with the reviewer on this issue and acknowledge that MotSASi limitations were poorly discussed, for example in its relation with AlphaFold and alternative structure prediction tools. Therefore, and according to the reviewer's suggestion we have in the discussion of the revised version of the manuscript included a whole new paragraph discussing the tool's limitations. As suggested, we have, in this section, specifically acknowledged that AF2 has challenges in disordered regions (as already mentioned), and discuss potential improvements using AlphaFold3 and other alternative structure prediction tools (e.g., RoseTTAFold, ESMFold). Moreover, we have also rewritten the statements about MotSASi outperforming AlphaMissense, focusing on its specific strengths, and the areas where both tools can improve.

Specifically, we added in the revised version the following paragraph (on Page 28 (Lines 640-645)): "Moreover, it has been documented that AF2 still struggles to accurately predict the conformation of intrinsically disordered protein regions, where, as previously mentioned, several SLiMs occur [43]. These cases might also benefit from AF3. Finally, it is worth mentioning that although MotSASi's pipeline is currently based on AF2, alternative structure prediction tools such as RoseTTAFold or ESMFold among others, could also be used."

And on Page 31 (Lines 707-711) "However, it is essential to consider the broader context: while MotSASi is a more suitable tool for assessing variants located within SLiMs, due to its specific

modeling of the underlying biological hypothesis, AM remains an excellent bioinformatics prediction tool with a considerably broader scope.”

Specific Answers to Reviewer #2

General Comment: SLiMs are important and understanding consequences of mutations and how they affect cellular wiring may be informative for molecular disease underpinnings and development of treatment. Molecular and especially structural studies have lagged behind as these modules can be difficult to capture, in part due to their intrinsically disordered nature. The manuscript is timely as novel methodologies like AF2 enable modelling of these interactions, and it is crucial to find out how well predictions of specificity on such models work. The manuscript is well-written, and easy to follow.

We thank the reviewer for his/her positive comments highlighting that the manuscript is timely, well-written, and easy to follow.

The main limitation is that the work only compares predictions on experimental structures vs. AF2 models, but not to experimental data such as sequences and affinities from phage display. The consensus patterns are by necessity limited in accuracy, and clinical data, while very interesting for applications, is not systematically available. Further, it appears that a large fraction of the contribution relates to the peptide alone, rather than the interaction (see below for details). While the peptide itself will of course, have important contributions, this might be misleading and also not the intention.

We thank the reviewer for noting the following two relevant issues:
1) First, that predictions are compared only to experimental structures and not other experimental data such as sequences and affinities from non-structural experiments. Although we agree that this comparison could help assess AF2 general capacity for predicting protein-peptide complexes, we believe that it is out of the scope of the present work. Our work, seeks not to thoroughly assess AF2 overall predicting capacity, but only to evaluate it as a tool for predicting SLiM receptor complexes, which are then used to further separate real SLiMs from false positives, and analyzed their variants in relation to clinical and variant allele frequency data. As already mentioned, our evaluation of AF2 models shows that in the present context, AF2 delivers adequate quality models, which are further validated by comparing their structural and clinical SAS matrices.

2) Second, We agree that, clearly, some relevant parts of the contribution are related to the intrinsic properties of the peptide, rather than the interaction. Following his/her suggestion, we have further analyzed this issue in the revised version (see below for specific modifications of the manuscript concerning this topic).

Major comments:

P8 “For genes with at least 10 pathogenic variants, the allele frequency threshold was determined based on the most frequent pathogenic variant reported in ClinVar.” - given how many random events affect allele frequency, such as population bottlenecks, this seems like a risky strategy. Is there a way to test how reliable this is? Are false positives or false negatives more problematic here? Would it be possible to consider sequences from great apes as controls?

This is a critical issue, and we agree that further clarification is required for non-specialist readers. In the context of rare disease diagnosis, it is essential to first, distinguish between different underlying disease mechanisms (dominant vs recessive). For dominant disorders, particularly those that are highly incapacitating, most pathogenic mutations arise de novo (i.e., they do not circulate in the general population). Consequently, their frequency in healthy population databases should be zero. There are exceptions, such as albinism, which is a mild condition. In such cases, pathogenic variants may be observed at higher frequencies. However, since these variants are well-characterized and multiple variants are typically available, our strategy accounts for this scenario. For recessive disorders, pathogenic variant frequencies tend to be higher. However, for most well-characterized diseases, the most frequent pathogenic variants are well known, and our strategy contemplates this fact.
Our strategy is widely implemented in clinical settings and follows a highly conservative approach, meaning that it prioritizes minimizing false positives, rather than maximizing sensitivity. Specifically, it only allows for the classification of high-frequency variants as benign but does not classify any variant as pathogenic. In other words, if a true pathogenic variant is defined as the true positive, the strategy inherently produces zero false positives, as it does not label any variant as pathogenic. The primary risk associated with this approach is the potential misclassification of a true pathogenic variant as bening (false negative). However, given the extensive variant frequency data available, this risk is considered negligible, or even nonexistent.

Figure 2: What are the units here?

We thank the reviewer for this comment. We have now added the units (kcal/mol) to figure 2.
The revised Figure legend now reads (Page 17, Lines 390-395):

“

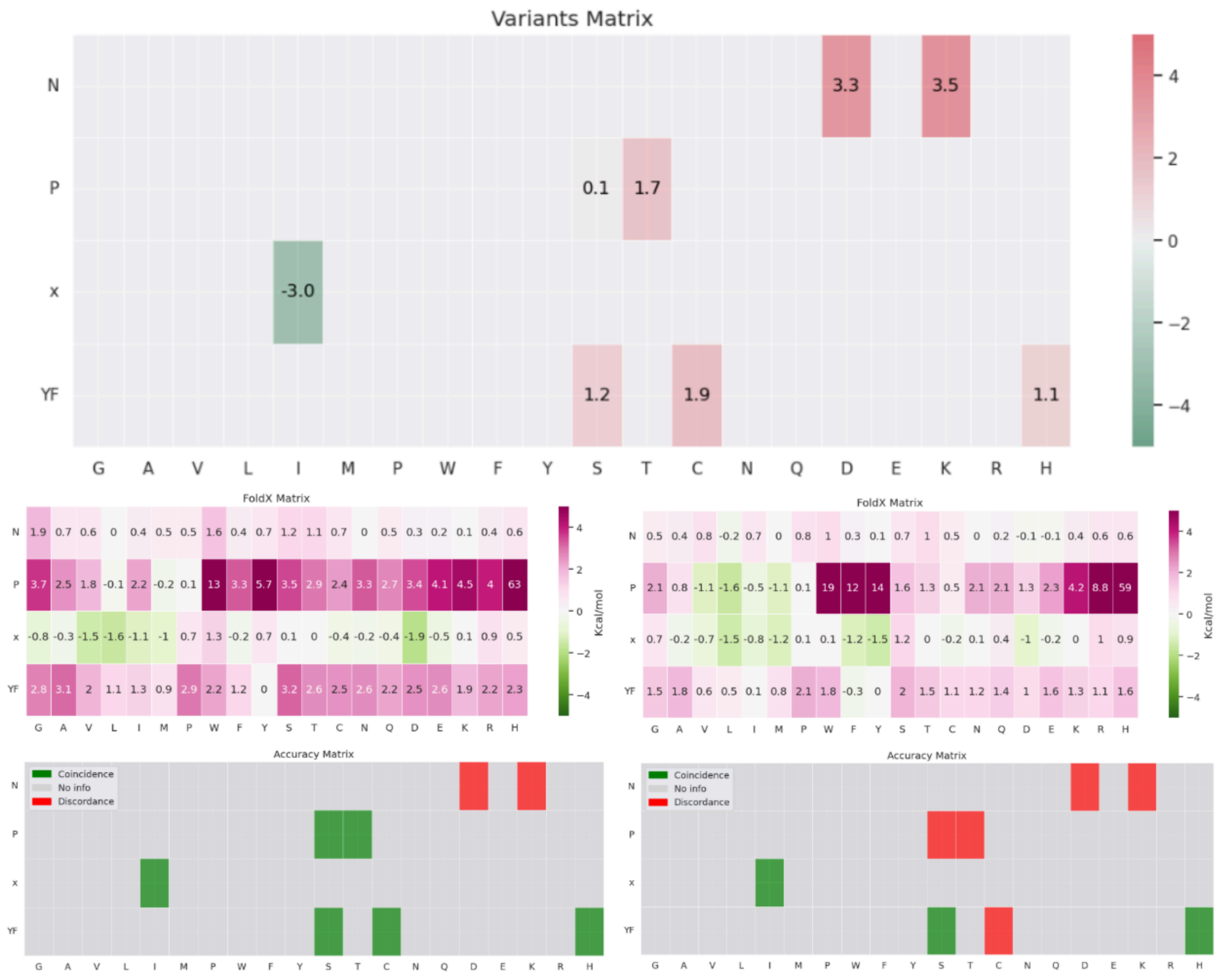

Figure 2. Comparison of the observed variant pathogenicity matrix (upper panel, expressed in ClinVar confidence units, as described in the Methods section), with the FoldX stability matrices (middle) for the correct (left panel) and incorrect AF2 models (right panel), with stability values expressed in kcal/mol. The lower panel summarizes the coincidences and inconsistencies between observed ClinVar and gnomAD variants and FoldX matrices.
”

It is interesting that even the incorrect models achieve fairly high accuracy. What can we learn from this? To be the devil's advocate, would it be enough to study the peptide in isolation? In other words, are most of the effects you find those on the monomer conformation? Of course these also affect whether there is an interaction, but it would be very nice if your work could also assess how much the domain side actually contributes? Is this largely a limitation of the tool used (FoldX), and have you explored alternatives?

We appreciate this insightful comment, which underscores the importance of understanding the thermodynamic contributions to the binding event. While we can manually assess whether a motif is correctly positioned by comparing it against crystallographic structures in the PDB, it is

crucial to acknowledge that AlphaFold2 implicitly accounts for thermodynamic principles when generating its predictions. This suggests that even when a motif is incorrectly positioned, its sequence, composition, and physicochemical properties may still contribute to a biologically relevant binding interface and underlying peptide conformation. In this scenario, variants that significantly affect intrinsic peptide structure (see the Proline examples mentioned below) or that alter its interaction with the receptor even if wrongly positioned, would result in a correct prediction.
In the revised version of the manuscript, and as suggested, we have further commented on this topic on Page 17 (Lines 397-404), by adding to the following paragraph: “Interestingly, although the structures and positioning of the SLiM relative to the receptor differ completely between the two models, the matrices still display some similarity. This observation arises from the inherent properties of residues and their general impact on protein structure (e.g. substituting a rigid Proline at row 2, as shown in the example of Figure 2, is typically more destabilizing than mutating the degenerate residue in row 3). Moreover, this effect is implicitly incorporated into AlphaFold2’s modeling of the binding event, as it inherently considers the underlying thermodynamic principles.”

The suggestion of looking more closely at the receptor (or domain) side is also very interesting, since variants in the receptor could negatively affect the SLiM binding, and lead to disease. Interestingly, if a binding affecting variant is present in the receptor, it would affect all receptor SLiM interactions, possibly leading to a different phenotype (and disease) when compared to those variants found in a particular SLiM. This interesting topic is, however, out of the scope of the present work. Nonetheless, and according to the reviewer’s suggestion, we have added the following paragraph on Page 30 (Lines 674-680): “Another interesting avenue for future research regarding variant pathogenicity assessment involves the receptor (or domain) side of the SLiM–receptor interaction. Variants occurring on the receptor side may also disrupt SLiM binding and contribute to disease. Notably, if a variant negatively affects binding at the receptor level, it could impair interactions with all SLiMs targeting that receptor—potentially altering the entire interaction network. This may lead to a distinct phenotype (and disease manifestation) compared to variants located within individual SLiMs.”

Within FoldX, why do you use PositionScan and not AnalyseComplex (https://foldxsuite.crg.eu/command/AnalyseComplex) which is for interface analysis?
In this context, it is worth noting that the label “interaction energy” is misleading.

We appreciate this comment and acknowledge that our method description was not accurate.
This issue has been clarified in the revised version of the manuscript. Indeed, we used both commands since they allow insight into slightly different effects of the SAS impact on the complex. When constructing the FoldX matrix, we utilized the PositionScan command to assess the intrinsic conformational stability and how the SAS impacts it. On the other hand, as noted by the reviewer, the AnalyseComplex command was employed to determine the SLiM-receptor interaction (i.e., binding) energies, both for setting the threshold value and assessing the quality of AF2 models. In the revised version of the manuscript, on Page 8 (Lines 201–211), the

Methods section describing the FoldX Free-Energy Change calculations has been revised as follows:

"

FoldX Free-Energy Change calculations

During the pipeline execution and SAS matrix generation, the stability Gibbs free-energy change (△△G) for each missense variant in the SLiM was calculated using FoldX, using the corresponding motif-domain complex structure (derived either from PDB or AF2). Calculations were performed using the PositionScan command. The resulting △△G values for each SAS in each motif class are presented as structural stability free-energy substitution matrices. When multiple structures were available for a given complex, the matrix values represent the average △△G across all structures.

To determine the SLIM-receptor interaction energy, establish the corresponding threshold, and further filter the AF2-generated models, we used the FoldX AnalyseComplex command across the different AF2 models.

"

https://doi.org/10.1002/anie.202213362 looks relevant in the context of this work and should perhaps be included.

We thank the reviewer for pointing out this nice work. We have carefully read the corresponding work and commented on it in the revised version of the manuscript. The corresponding work corresponds to reference 26 in the revised version of the manuscript. The corresponding comment (on Page 6 (Lines 132-135)), reads: "Moreover, recent studies have shown that AF2's has already surpassed the ability of other docking programs in predicting and ranking peptide-protein complexes, and that it allows identifying those peptides that might have the highest binding affinity and their structure [26]."

Generally, the figure legends need more detail, including for supplementary figures. For example, for Suppl. Figure S1, what are RC, FxC, FIC? In Suppl. Figure S4, where exactly is the source for "previously classified" cases? Reference?

We agree with the reviewer and thank her/him for pointing out this issue. Therefore, in the revised version of the manuscript, and supplementary information. We have worked hard to modify the Figure legends, providing more detail and better explanations to allow the reader to better comprehend each Figure.

For example, we modified the SI Figure legends as follows (figures are included for direct evaluation of the changes):

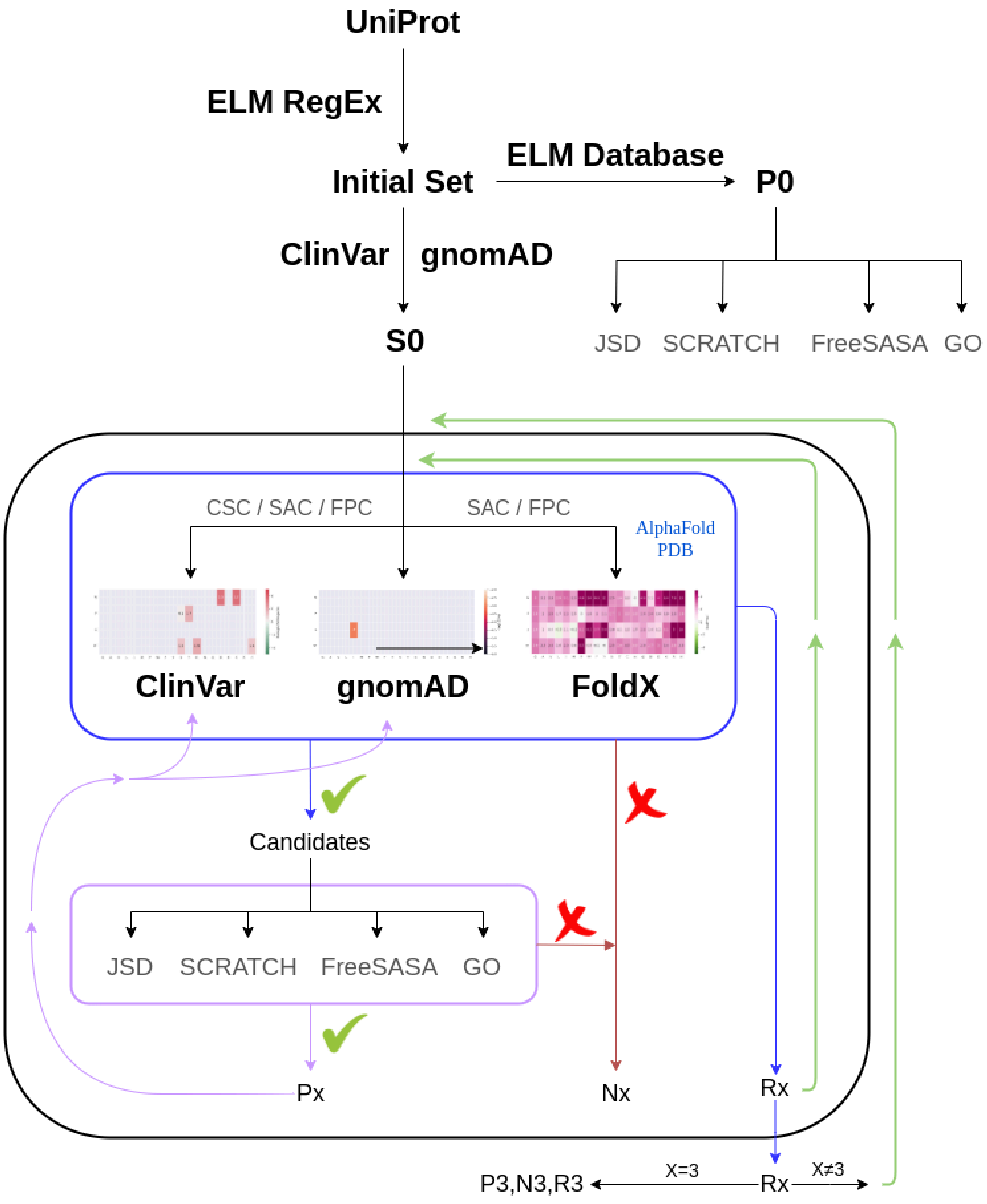

Figure 1 SI: Flowchart of the MotSASi pipeline, integrating crystallographic structures from the PDB and predictive models from AlphaFold2. CSC = Clinical Significance Cycle (x=1), SAC = Structural Analysis Cycle (x=2), FPC = Flexible Position Cycle (x=3), JSD = Jensen-Shannon Divergence, P = Positive set, N = Negative set, R = Remaining set. The CSC, SAC, and FPC

cycles occur sequentially. Candidates in the S0 and Rx sets are assessed using ClinVar and gnomAD matrices in all three cycles, whereas candidates in the R1 and R2 sets are evaluated against the FoldX matrix exclusively in the SAC and FPC cycles.

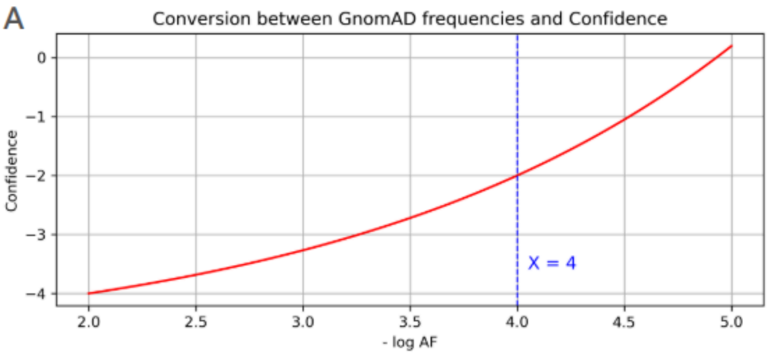

$$\text{Confidence Score} = \frac{1}{3} \cdot \left(\sqrt{3}\right)^{\log(\mathrm{AF})} - 5$$

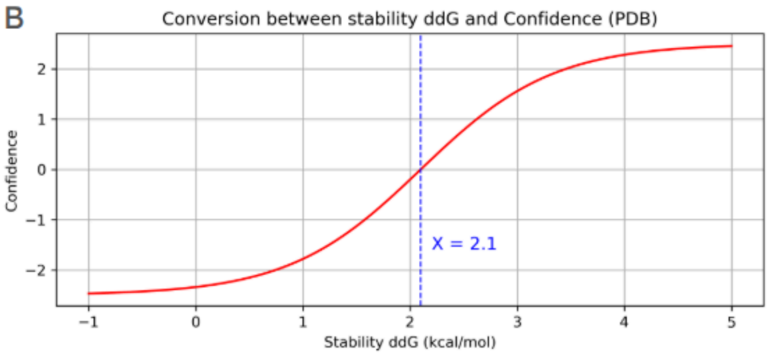

$$\text{Confidence Score} = \frac{5}{1 + e^{\ln\left(\left(\frac{2}{3}\right)^{4}\right)\cdot(x-2.1)}} - 2.5$$

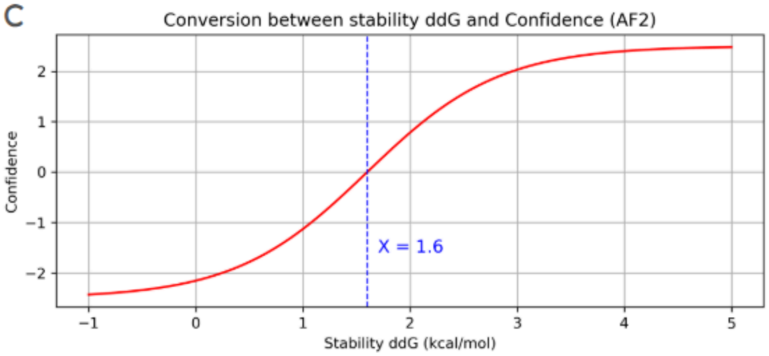

$$\text{Confidence Score} = \frac{5}{1 + e^{\ln\left(\left(\frac{2}{3}\right)^{4}\right)\cdot(x-1.6)}} - 2.5$$

Figure 2 SI: Plots of functions and their corresponding formulas for (A) gnomAD, (B) PDB-derived, and (C) AF2-derived Confidence Scores.

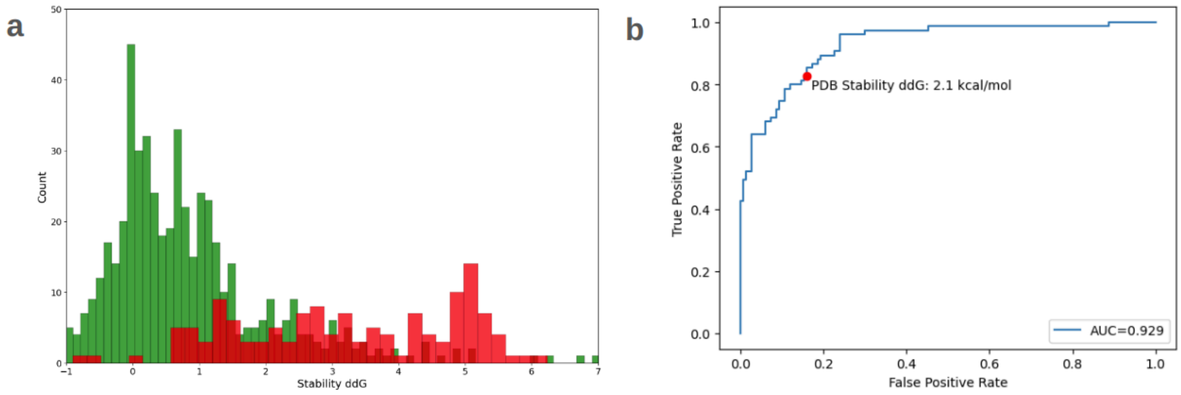

Figure 3 SI: (a) Histogram displaying the ClinVar and gnomAD variants used to define the △△G stability threshold in (kcal/mol). Pathogenic variants are indicated in red, while benign variants are shown in green. (b) ROC curve (AUC = 0.929) generated during the △△G threshold determination process for PDB crystallographic structures. The red dot marks the threshold value of 2.1 kcal/mol, corresponding to a sensitivity of 83% and a specificity of 84%.

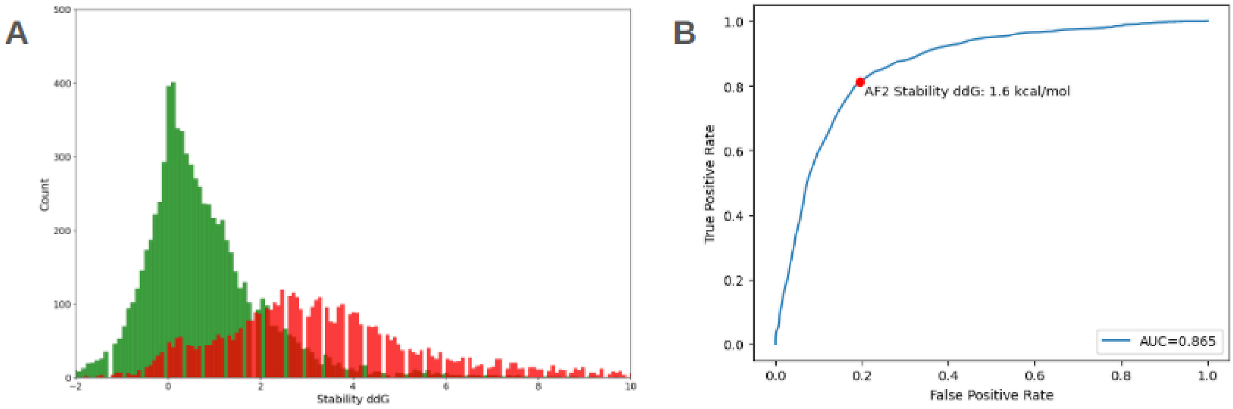

Figure 4 SI: (a) Histogram of △△G stability values (in kcal/mol) calculated on models generated by AF2 for the same ClinVar and gnomAD variants previously classified as tolerated or non-tolerated in PDB-deposited crystallographic structures, based on the previously mentioned 2.1 kcal/mol threshold. Non-tolerated variants are shown in red, while tolerated variants are shown in green. (b) ROC curve (AUC = 0.865) generated during the process of determining the △△G cutoff value for AF2. The red dot marks the cutoff value of 1.6 kcal/mol, corresponding to a sensitivity of 81% and a specificity of 80%.

p9 describes how ClinVar evidence is “scored”. A common minimum standard is “at least one star”. While in this work, the stars are counted, it is unclear what would happen for a variant with 5 pubs saying patho and 5 saying benign, this might still add up to 1?

We appreciate this comment and have improved the handling of ClinVar database data treatment in the revised version of the manuscript, while also explaining more clearly how the evidence is scored. Consistently, we added the following statement in the revised version (on Page 9 (Lines 215-223)): “Missense variants classified as "Benign", "Benign/Likely benign", "Likely benign", "Pathogenic", "Pathogenic/Likely pathogenic" or "Likely pathogenic" were obtained from ClinVar. The clinical significance of these variants was converted into confidence scores, assigning 1 point per review status star and 0.1 points per supporting submission. For example, a variant classified as "Pathogenic" in ClinVar with a two-star review status and three supporting submissions would receive a confidence score of +2.3. Conversely, a variant classified as "Benign" with a one-star review status and one supporting submission would receive a confidence score of -1.1.”

Did you consider other predictors beyond AlphaMissense, e.g. ESM1v?

No, we didn't consider other predictors beyond AlphaMissense since the work focus is not a comparison of different methods for predicting variant effects. In any case, this comment is related to the comment from pages 8-10 (from the response to reviewers file) of reviewer 1, and according to both reviewer suggestions, we have included a comment on the potential use of other predictors in the discussion of the revised version of the manuscript.

Specifically, on page 23 (Lines 519-524) we added the following statement: “In this context, we sought to compare MotSASi with another method that relies on structural information to train its algorithm. Given its recent success, we selected AlphaMissense (AM) as the candidate, although other bioinformatic prediction tools recommended by ClinGen for assessing clinically relevant variants—such as REVEL, BayesDel, MutPred2 and VEST4—could also have been used [42].”

Minor comments
P4, “on the contrary” - flow is strange, rephrase

Type has been corrected on Page 4 (Lines 78-80): “In contrast, since most human (as well as other species) protein sequences are known, thousands of SLiMs have been bioinformatically predicted and await further scrutiny[4].”

P13 “kery to our approach” - typo, should be key?

Type has been corrected on Page 13 (Lines 328-329): “Key to our approach is the accurate positioning of the SLiM within its corresponding binding site (Figure 1A, left panel).”

P15 “classified as reproducible” what does this mean? Technically reproducible? Or finding the same conformation as in the xtal structure? Yet a third option?

We have revised the figure legend where this issue appears on Page 15 (Lines 354–362) to improve clarity.

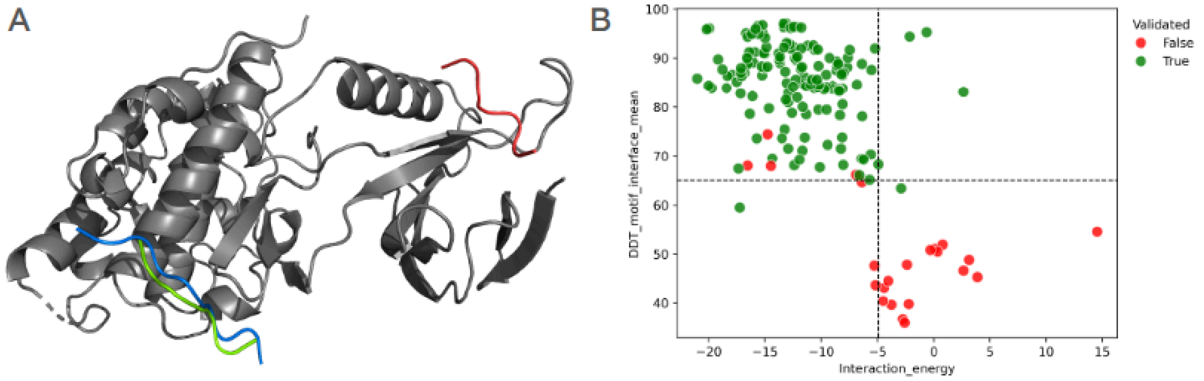

Figure 1. (A) Crystallographic structure (pdbid 1UKH), depicting the interaction between the DOC_MAPK_JIP1_4 motif (in blue) of mouse JIP1 (UniProtID: Q9WVI9) and the Pkinase domain (in grey) of human MAPK8 (UniProtID: P45983). Two AF2 predictions for the motif are shown: a correct prediction (in green), and an incorrect prediction (in red). (B) Scatter plot showing the relationship between the mean pLDDT of the motif residues (y-axis), and the motif-receptor interaction energy in kcal/mol (x-axis). Each dot represents an individual AF2 model: green dots indicate models manually classified as correct, while red dots denote models classified as incorrect. Dashed lines indicate threshold values selected to distinguish between correct and wrong AF2 predictions.

P17 “Summarizying” typo

Type has been corrected on Page 18 (Line 421): “Summarizing both analyses, it is clear that, taken together, the motif residues’ mean pLDDT, the SLiM-receptor interaction energy, and the comparison of the FoldX and clinically significant variant matrices, allow for the selection of the best AF2 models.”

P12 + Fig. S1 - Do the clinical significance cycle and structural analysis cycle happen simultaneously (Fig. S1) or one after another (text on page 12)?

The Clinical Significance Cycle and Structural Analysis Cycle occur sequentially. To enhance clarity, we have thus reformulated Figure S1.

New Figure and legend are as follows:

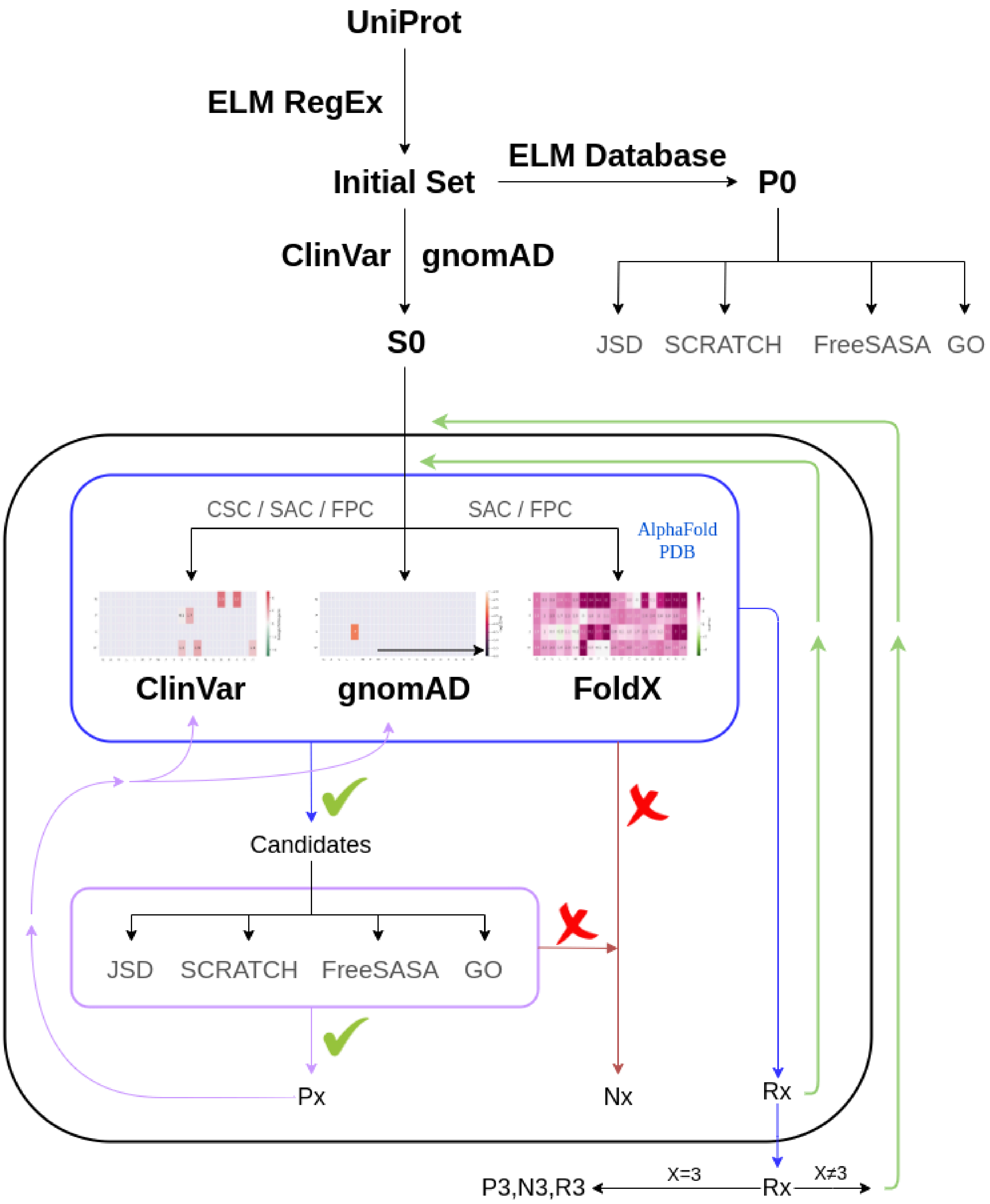

Figure 1 SI: Flowchart of the MotSASi pipeline, integrating crystallographic structures from the PDB and predictive models from AlphaFold2. CSC = Clinical Significance Cycle (x=1), SAC = Structural Analysis Cycle (x=2), FPC = Flexible Position Cycle (x=3), JSD = Jensen-Shannon Divergence, P = Positive set, N = Negative set, R = Remaining set. The CSC, SAC, and FPC

cycles occur sequentially. Candidates in the S0 and Rx sets are assessed using ClinVar and gnomAD matrices in all three cycles, whereas candidates in the R1 and R2 sets are evaluated against the FoldX matrix exclusively in the SAC and FPC cycles.

Fig. S5 - again, no details. What is this motif shown as an example? I don’t think it’s in the main text, either

The aim of this figure is to illustrate that there is no substantial difference between the matrices derived from PDB and AF2, as mentioned in Line 367 of the revised manuscript. Nonetheless, we have added the requested specifications in the figure legend, which now states:

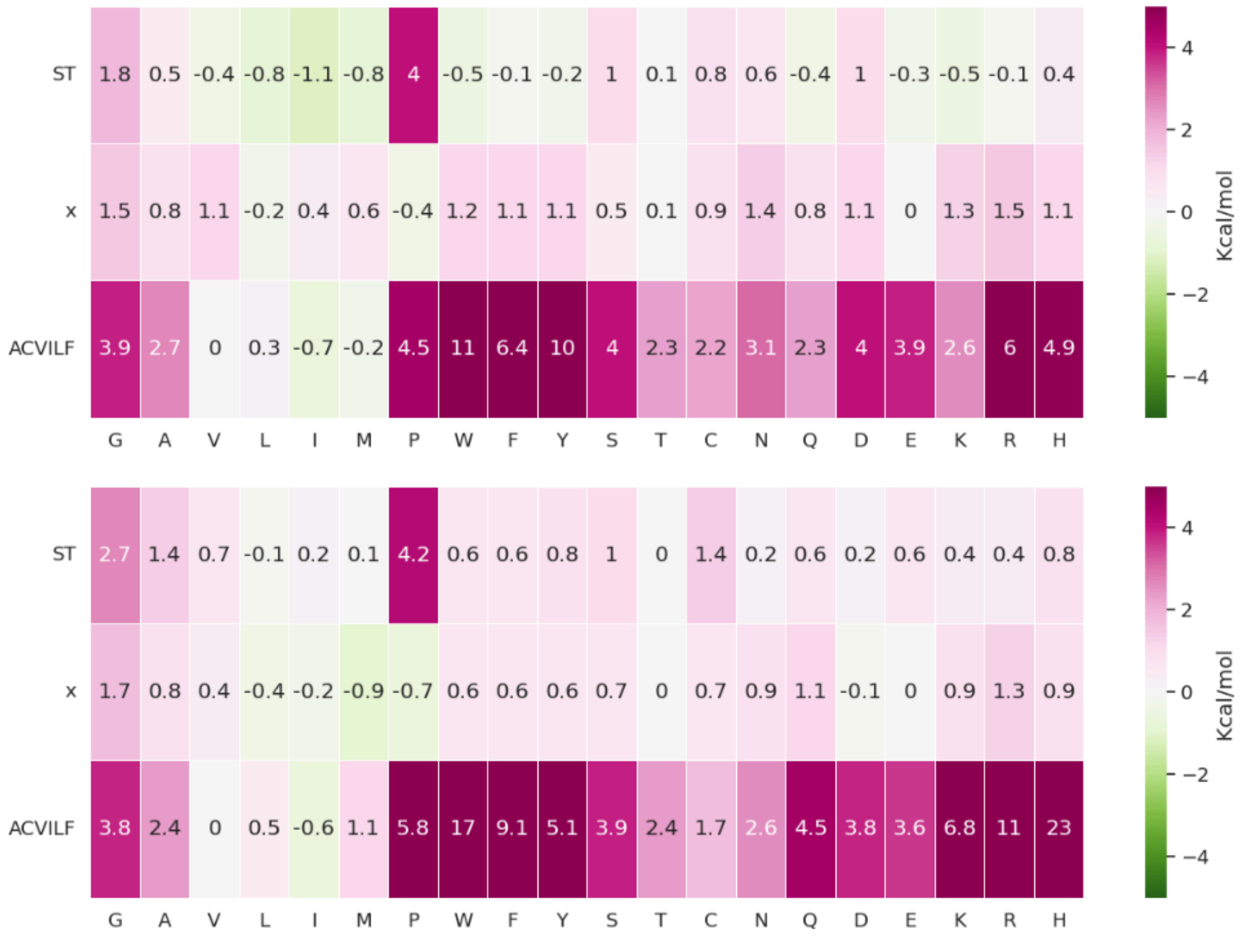

Figure 5 SI: Comparison of structural (stability ΔΔG) SAS matrices obtained using either the crystallographic structure deposited in the Protein Data Bank (PDB) (upper panel) or the SLiM-receptor structure modeled by AlphaFold2 (AF2) (lower panel) for the LIG_PDZ_Class_1 ELM motif class (regular expression: [ST].[ACVILF]$).

P15 - If I am reading this correctly, they get the Perason’s R of 0.65 with the error of 0.22 for the FoldX predictions on crystals and on AF2. This is quite low? Is this because some AF2

predictions were way off? + As a figure for this paragraph we get Fig. S5, which is one individual case out of many, and we are supposed to inspect it visually.

This is an interesting point raised by the reviewer, and accordingly we have looked with further detail into it. Upon inspecting the full matrices, we identified a specific pattern: while FoldX yields linearly correlated values between calculations performed on PDB and AF2 structures within the -2 to 6 kcal/mol range, the linearity is lost for higher values—where the deleterious effect is unquestionable—and likely due to structural deviations in AF2 models affecting extreme stability predictions. Nonetheless, the strong monotonic relationship remains.
In the context of the observed behavior, in the revised version we conducted a non-parametric Spearman correlation test, which is more appropriate for detecting monotonic associations when linearity is not preserved in the whole range. The resulting Spearman correlation coefficient was 0.73 ± 0.15, demonstrating a stronger agreement between PDB- and AF2-derived FoldX calculations than Pearson's R suggested (and noted by the reviewer). These findings confirm that AF2 remains a highly valuable alternative for stability predictions, particularly for moderate stability changes, while maintaining overall reliability even in more extreme cases. These findings are now reflected in the revised manuscript on Page 15 (Lines 369-374) where it states: "First, we computed the Spearman correlation coefficient between the ΔΔG values obtained with either matrix, yielding a value of 0.73 +/- 0.15. We selected the non-parametric Spearman test because, while a linear correlation holds within the -2 to 6 kcal/mol range, it breaks down at higher values—where the deleterious effect is unquestionable— of important note is that a strong monotonic relationship remains."

Fig. 2 has a bad resolution (difficult to read)

We have modified Fig 2 to improve its resolution in the revised version of the manuscript.

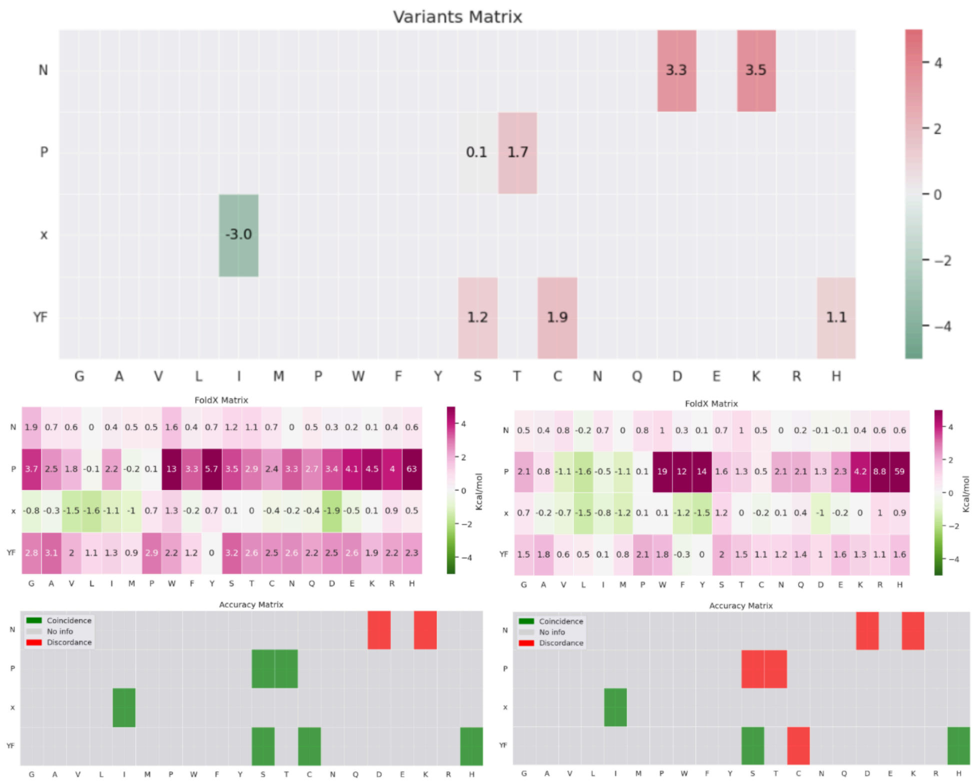

Reviewer #3: In “Integrating AlphaFold2 Models and Clinical Data to Improve the Assessment of Short Linear Motifs (SLiMs) and Their Variants’ Pathogenicity” the authors present an improved prediction method for SLIMs with Mendelian variants. The project holds promise but the manuscript lacks important details, especially in the method section. This makes it difficult to fully evaluate the work and review the manuscript. The authors are encouraged to consider the following examples and apply them throughout their manuscript:

We thank the reviewer for his/her positive comment on our manuscript, highlighting that the project holds promise. We also agree with the reviewer that we need to provide more detail, especially in the Methods section.

1. Some things are described in methods but not presented in results. For example:
a. For the SLIM predictions described in methods, what was found? This could at least be presented in the supplementary materials.

We thank the reviewer for the comment. We would like to emphasize that the results of motif predictions in the whole human proteome are summarized in Table 1, which includes both negative and positive high-confidence candidate counts.

b. For some SLIMs, experimental models were found and used while for others AlphaFold models were used, how many of each? This could at least be in the supplementary materials.

We agree with the reviewer that this is relevant information. Indeed, in the original version of the manuscript, we stated on page 20, line 398, that we analyzed 27 motif classes with crystallographic structures deposited in the PDB, and 24 motif classes based on AF2-generated models. Additionally, this information is provided in Table 1 under the PDB and AF2 columns. In any case, and to strengthen this point and avoid further misunderstandings, We have explicitly added this information again in the methods section of the revised version of the manuscript.

Specifically, in the methods section we added the following sentences (on Page 7, Lines 153-155): "In total, 24 motif classes from the former group and 27 from the latter were studied. A subset of the former group (17/24) was used in the AF2 motif-domain complex structure prediction validation procedure."

2. Methods are not sufficient. For example:
a. The AlphaFold2 (AF2) models: it is mentioned that the best complex is selected and the mean plddt for best complexes is 65, which is in the low confidence range. What are the confidence levels of the SLIMs and their models – this should be included in the manuscript.

We thank the reviewer for pointing out this issue. In response, we have specified the mean pLDDT values for all the motif residues and the interaction energy values obtained for both correct and incorrect models. Additionally, we have tested the statistical significance between the means of both groups. In the revised version the following results describing the topic has been included (on Page 14, Lines 339-342): "As shown in Figure 1B (right panel), correct models exhibit a higher mean pLDDT for AF2-predicted SLiM residues (82.8 ± 10.0 vs. 40.9 ± 14.0, $p < 1 \times 10^{-39}$) and a lower interaction energy between the receptor and the SLiM (-12.1 ± 4.1 kcal/mol vs. -3.1 ± 6.6 kcal/mol, $p < 1 \times 10^{-15}$)."

b. Further, for the AF2 models, the method section is very brief yet the results state that "analyzed several parameters of the AF2 models to assess their ability to discriminate between correct and incorrect SLiM positions" but how these analyses were performed is not described.

We agree with the reviewer that methods were not described with enough detail (a point also raised by the other reviewers). Therefore, and according to his/her suggestion we have in the revised version of the manuscript significantly expanded the method description. In particular, as suggested, we have better explained the parameters and details used in the AF2 calculations, and also how the best complexes were selected.

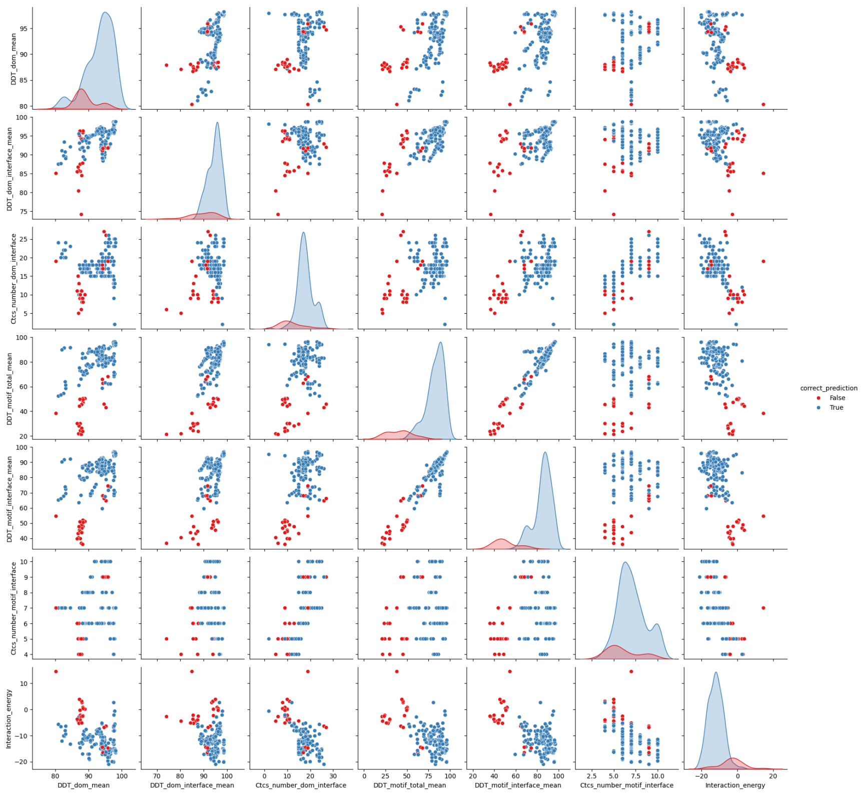

3. Limited result presentation. For example:

a. Instead of showing a few select examples to claim model accuracy, what does the overall data show?

According to the reviewer's suggestion in the revised version of the manuscript, we have explicitly, and clearly explained what the overall data shows in relation to MotSASi's model accuracy. As stated in the answer to the next point, in the revised version we have further explained how overall accuracy was computed, asn it is explicitly stated after presenting the mentioned examples. See below for explicit description of the added paragraph.

b. The results state a global accuracy metric was calculated and that the percent accuracy was 74% for correct models and 63% for incorrect models. How this was performed is not described and if there is a statistical difference between the correct and incorrect models is also not mentioned. This must be improved.

We thank the reviewer for noting this point. Accordingly, in the revised version of the manuscript, we have clearly explained how the accuracy was computed in each case. We also measured if the difference is statistically significant.

Consistently, in the revised version we added the following statement on Page 18 (Lines 412-420). “As a result, we performed a broader evaluation by aggregating all observed variants within the motif classes considered for the validation process. We calculated a global accuracy metric for those AF2 models that passed, and those that did not pass the confidence and interaction energy thresholds (as established in the previous section). AF2 models that met both

criteria achieved an overall accuracy of 77.2%, while models that did not meet the thresholds achieved an overall accuracy of 58.2%. This difference was statistically significant, as shown by the proportions comparison test (p-value < 0.0005).”

c. The figure legends need to provide more detail to describe what is shown. If an example of a SLIM is shown, include the reg ex pattern.

We agree with the reviewer that Figure legends need more detail (as also noted by the other reviewer). Moreover, adding the regular expression pattern associated with the motif in each figure is a very good idea. Therefore, in the revised version of the manuscript, we have significantly improved the figure legends, and explicitly added the corresponding Regular Expression patterns. Specifically, the regular expression was added to the legend of Figure 5 SI.

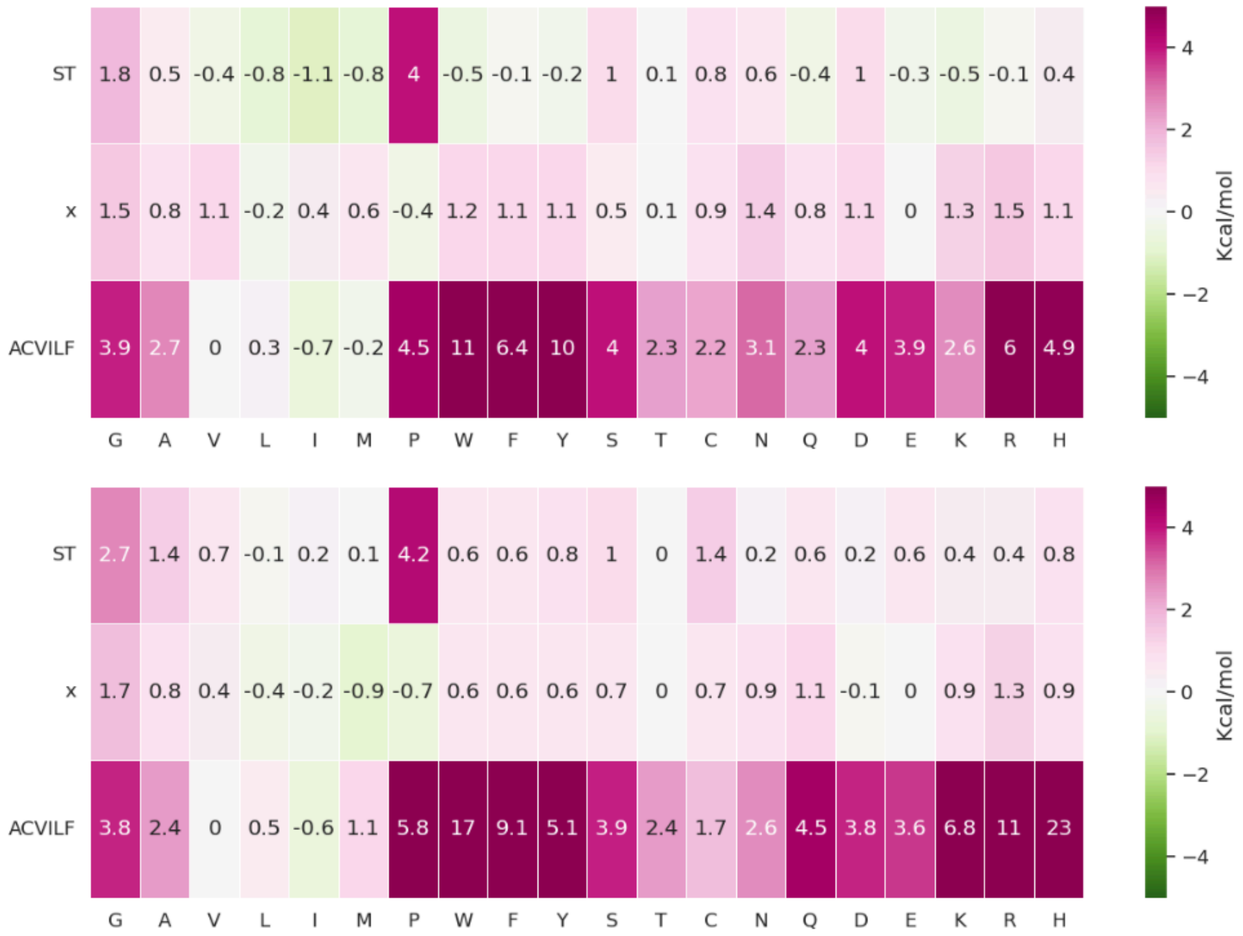

Figure 5 SI: Comparison of structural (stability ΔΔG) SAS matrices obtained using either the crystallographic structure deposited in the Protein Data Bank (PDB) (upper panel) or the SLiM-receptor structure modeled by AlphaFold2 (AF2) (lower panel) for the LIG_PDZ_Class_1 ELM motif class (regular expression: [ST].[ACVILF]$).

4. More detail on the comparison with AlphaMissense is needed. What dataset was used? How was it selected?

According to the reviewer's suggestion, in the revised version of the manuscript, we have provided more detail on the comparison with AlphaMissense, also better explaining which dataset was used and how it was built. Specifically, we added the following section (on Page 13, Lines 308-316):

"

Comparison Against AlphaMissense: Evaluation Pipeline Design

To perform a comparison with the bioinformatic pathogenicity predictor AlphaMissense, we developed a modified pipeline. In each cycle and iteration, a subset of 20% of ClinVar pathogenic variants and gnomAD benign variants, identified in ELM instances (true positives) or newly detected high-confidence motifs, is set aside to form an independent evaluation set. These variants are excluded from the pipeline and subsequently used to assess the performance of the MotSASi SAS matrices in comparison with AlphaMissense predictions, utilizing metrics such as accuracy, F1-score, and Matthews Correlation Coefficient (MCC).

"

5. More context for the ACMG standards and aims is needed.

According to the reviewer's suggestion we have provided, in the revised version of the manuscript, more context related to the ACMG standards and the aims concerning the present work. Specifically, we added the following paragraph (on Page 28, Lines 646-655): "The high-confidence SLiM candidates and missense substitution matrices provided by MotSASi contribute to the clinical genomics community with tools to refine ACMG guidelines for variant interpretation [44]. These widely adopted standards in clinical molecular diagnostics emphasize that the interpretation of clinically relevant variants must be conducted according to a set of criteria, including familial segregation, variant allele frequency, molecular effect, and others, which are implemented in the form of 28 different alphanumeric labels. These criteria, or labels, are commonly referred to as the ACMG labels/criteria for variant annotation. Among these, one particularly relevant to MotSASi is the pathogenic and moderate PM1 label."
