# Supplementary material for: Integrating AlphaFold2 models and clinical data to improve the assessment of Short Linear Motifs (SLiMs) and their variants’ pathogenicity
Source: PLoS Comput Biol. 2025 Aug 4;21(8):e1012829. doi: 10.1371/journal.pcbi.1012829 (PMC12338786; doi:10.1371/journal.pcbi.1012829)
Supplement: Attachment — Submitted filename: MotSASi2.0_Response_to_Reviewers_2.pdf [file pcbi.1012829.s005.pdf]

Buenos Aires, May 23, 2025

Ref Ms# PCOMPBIOL-D-25-00157

Dear Dr. Mohammad Sadegh Taghizadeh, Ph.D., Academic Editor, & Dr. Ilya Ioshikhes, Section Editor
PLOS Computational Biology

We are pleased to submit the second revised version of our manuscript, "Integrating AlphaFold2 Models and Clinical Data to Improve the Assessment of Short Linear Motifs (SLiMs) and Their Variants' Pathogenicity" by Brunello et al.

We have carefully reviewed and addressed all the new comments and suggestions provided by the reviewers, which have been invaluable in enhancing the quality and clarity of our work. Accordingly, we have revised and rewritten the manuscript as needed. A detailed, point-by-point response to the reviewers' comments, along with a summary of the changes—highlighted in red in the revised manuscript—is provided below.

We appreciate your time and consideration.

Best Regards,

Bioq. Franco Gino Brunello
Department of Biological Chemistry, Faculty of Exact and Natural Sciences, University of Buenos Aires (FCEyN-UBA), and Institute of Biological Chemistry (IQUIBICEN), National Scientific and Technical Research Council (CONICET)

Specific answers to the Reviewer #1

Reviewer #1: The authors properly addressed all the comments and made the github repo available.

We sincerely thank Reviewer #1 for his/her thorough evaluation of our revised manuscript and for acknowledging our efforts in addressing his/her previous comments. We are pleased to hear that the revisions were satisfactory and that the availability of the GitHub repository met his/her expectations.

Specific Answers to Reviewer #2

Reviewer #2: The authors have thoroughly worked on the manuscript and addressed most of my comments. One remaining issue is the statement that, in clinical assessment, false negatives would be less concerning than false positives. Missing true positives clearly is a problem, especially if diagnosis is actionable (e.g. BRCA variants). That said, given that computational results alone will currently not be used for diagnosing, perhaps this point is not as critical. I would nevertheless recommend phrasing impacts of false positive/negative carefully - for any given patient, this is a massive impact on their quality of life.
I would like to include this paper: https://doi.org/10.1038/s44320-023-00005-6 which I believe would also be relevant for the authors to consider.

We sincerely thank Reviewer #2 for his/her constructive feedback,and for recognizing the thorough work we have done in revising the first version manuscript.

We also appreciate the valuable suggestion regarding the importance of carefully considering the impact of false positives and false negatives in clinical assessment. We agree with the reviewer that in the current version, the statement, “in clinical assessment, false negatives would be less concerning than false positives,” is biased. And thank him/her for correctly highlighting that missing true positives is a problem, especially if diagnosis is actionable (e.g. BRCA variants). Therefore, and according to his/her suggestion of specifically phrasing the impact of false positives/negatives carefully, we have in the revised version of the manuscript rewritten the statements related to the relevance of false positives and false negatives in a clinical setting. We have also, according to his/her suggestion, better clarified the clinical context in which our computational predictions are intended to be used. Specifically in page 33 we state that:

Lines [710-723]: “Analysis of the confusion matrices generated by each method revealed that, while AM provides more accurate predictions for pathogenic variants in the test set, it also produces many false positives. It is important to emphasize that in clinical genomics, both types of misclassifications are problematic, but in different contexts. In the case of rare diseases diagnostics, where usually a clinical diagnosis is already available, false negatives are generally preferable to false positives and it is more prudent to withhold reporting a candidate variant until

stronger evidence is available, rather than reporting it prematurely and risking subsequent retraction. However, false negatives also carry clinical risk, particularly when they lead to missed opportunities for timely intervention. This is especially critical in the context of actionable genetic findings—such as pathogenic variants in BRCA1 or BRCA2, which are included in the ACMG list of secondary findings—where early detection enables established preventive strategies aimed at significantly reducing morbidity and mortality [48].”

Furthermore, we are grateful for the reviewer’s suggestion to include the reference (DOI: 10.1038/s44320-023-00005-6), concerning the Systematic discovery of protein interaction interfaces using AlphaFold, which is directly related to our work. Accordingly, in the revised version of the manuscript, we incorporated this work as reference 27 in the Introduction Section, highlighting its insightful exploration of motif–domain interactions in the context of neurodevelopmental disorders, and the collaborative approach it represents. Specifically, the following statement is added:

Line [140-143]: “Importantly, AF2 has also been successfully applied to study domain–motif interactions in the context of neurodevelopmental disorders, although predictive accuracy was shown to decrease when full-length proteins containing the motif were used as input, highlighting the need for tailored fragmentation strategies [27].”

Reviewer #3:

Point 1) I don't find the improvements of this ms sufficient. There is still a disconnect between the methods and the results. The results are incomplete. It is briefly mentioned that most SLIMs are in disordered loops and perhaps why this method outperforms AlphaMissense. Can the authors better describe what characterizes the SLIMs in their data and what characterizes accurately and inaccurately predicted SLIMs for the two methods? How do GO terms help the prediction?

We thank the reviewer for carefully re-reading the manuscript and for the valuable suggestions that helped us further improve the clarity and depth of our work. Specifically, the reviewer raised three main points: (i) the need for a stronger connection between the methods and results sections, particularly through a clearer description of what characterizes SLiMs that are positively and negatively predicted by our method, MotSASi; (ii) a more detailed analysis of accurately and inaccurately predicted SLiMs, especially in comparison with AlphaMissense; and (iii) a deeper discussion on the role of GO terms in the prediction pipeline. We have addressed these points throughout the revised manuscript, incorporating additional analysis and figures to clarify each aspect. We believe these changes have significantly strengthened the manuscript, particularly by highlighting the distinct design principles behind MotSASi and how these contribute to its performance in predicting SLiM functionality.

i) Regarding the first point, we appreciate the reviewer’s concern about the connection between the methods and results sections, as well as the need for a more detailed characterization of how the SLiM selection methodology leads to the classification of motifs as either positive or negative. In response to this suggestion, we have revised the beginning of the “SLiMs analysis

and filtering pipeline" section in the Methods to better explain how the successive features computed for each SLiM—such as conservation, secondary structure, solvent accessibility, and GO term compatibility—guide MotSASi in selecting or discarding candidate motifs.

To further improve the connection between methods and results, we have added Supplementary Figure 7, which outlines both qualitatively and quantitatively how SLiMs candidates are progressively filtered throughout the pipeline, according to the different computed properties. This visual representation aims to make the logic of motif selection more transparent and to reinforce the alignment between our methodology and the resulting dataset.

The Figure, shown below, leads to the following analysis, presented in the revised version:

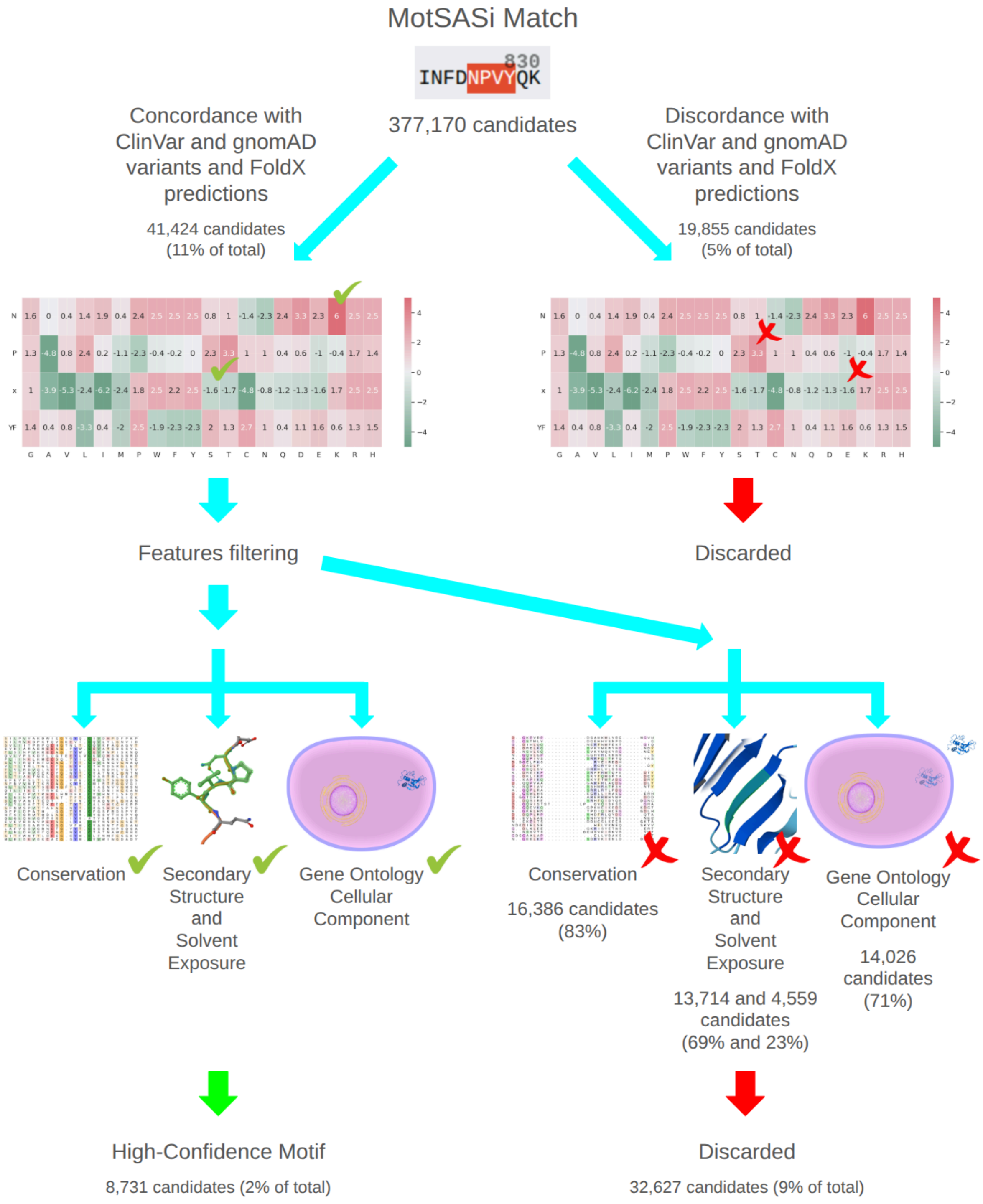

In the end of the methods section, we also underscored the positive predicted SLiMs characteristics required by MotSASI. Specifically, we added the following sentence:

Lines [324-327]: “All selected variants are located within motifs that satisfy the full set of structural, evolutionary, and annotation-based filters described in the pipeline, including

conservation, secondary structure, solvent accessibility, and GO term coherence with true positive motif class instances."

In the results section we focus specifically in the filtering steps underwent:

Lines [464-482]: "These results, together with the statistics presented in Fig 7 SI, allow for a more comprehensive analysis of the features that characterize accurately predicted, positively selected SLiMs, as well as those that are discarded during the filtering process. Starting from the total number of Regular Expression (RE) matches (considered as 100% of the potential SLiMs), a large fraction (84%) cannot be further analyzed due to the absence of clinically relevant variants. Among the remaining 16% of SLiMs that do contain such variants and can be processed through our pipeline, 79% are ultimately discarded—an expected outcome given the low specificity of REs. The primary reason for discarding candidate motifs is insufficient evolutionary conservation (83% of evaluated candidates), followed by discordant GO term annotations (71%), incompatible secondary structure (69%) relative to known instances of the motif class, and finally, limited solvent accessibility (23%). Notably, a given candidate may be excluded based on more than one of these criteria (e.g., being both poorly conserved and structurally buried). Conversely, the high-confidence motifs retained by MotSASi—representing approximately 2% of the total initial candidates—are those that exhibit strong coherence with the features observed in true positive motif instances. These include baseline levels of conservation, structurally compatible secondary structures (typically disordered), solvent accessibility, and consistent subcellular localization based on GO annotations."

In the discussion section, we have reintroduced this important consideration regarding the construction of the evaluation dataset:

Lines [757-762]: "Last but not least, it is important to acknowledge that the dataset used to compare both methods may be inherently biased in favor of MotSASi, as all tested variants were selected from motif matches that passed conservation, structural, and annotation filters. These filters align with typical SLiM characteristics—such as their location in disordered or loop regions—where AM has been previously reported to show lower performance."

ii) Concerning prediction accuracy and comparison with AlphaMissense (AM), we appreciate the reviewer's observation and agree that a deeper comparison is indeed valuable. In response to this suggestion, we have extended the comparative analysis between both methods, with particular emphasis on the specific cases where their predictions diverge. We now provide a more detailed characterization of the features that distinguish correctly and incorrectly predicted SLiMs by each method.

To further investigate the differences in predictive performance between MotSASi and AlphaMissense, we analyzed the potential sources of this discrepancy.
First, we included in the analysis those variants predicted as Uncertain by both methods, which provides insight into how each approach applies its classification thresholds. As mentioned previously, the main difference between MotSASi and AM lies in their treatment of benign

variants: AM fails to correctly classify approximately 25% of benign variants, whereas MotSASi shows a negligible rate of benign misclassification. When examining these misclassified benign variants by AM, over half are labeled as Uncertain, and the remainder are incorrectly classified as pathogenic. This suggests that part of the discrepancy in performance stems from the thresholding strategies used. AM applies a general threshold designed to work across all human proteins and their regions, while MotSASi uses a tailored threshold for SLiMs in the context of their interactions with protein partners.
The second relevant point concerns the intrinsic nature of SLiMs and their interaction interfaces, as reflected in their Regular Expression (RE) definitions. Typically, some RE positions are structurally constrained, allowing only a limited set of amino acids (which we refer to as “rigid positions”), while others are more permissive, allowing for a broader amino acid diversity (“flexible positions”). We separately analyzed prediction performance for variants located at rigid versus flexible positions. While both algorithms performed similarly on flexible positions (MCC: MotSASi 0.50 vs. AM 0.37), a much larger performance gap was observed for rigid positions (MCC: MotSASi 0.88 vs. AM: 0.41). These findings are consistent with the nature of the two methods: AM is a general-purpose predictor that focuses on the isolated variant-containing protein, without explicitly modeling its interactions, whereas MotSASI directly evaluates the variant effect within the context of the motif-domain complex. As rigid positions typically make direct contacts with the receptor, MotSASi’s use of structural modeling allows for more accurate prediction. In contrast, flexible positions often do not engage directly in binding, which may explain the comparable performance between both tools in those cases.

Specifically, on page 34 we added:

Lines [734-756]: “A deeper analysis of the accuracy differences between the two methods reveals two main contributing factors. First, upon further examination of the benign variants misclassified by AlphaMissense (AM), we found that over half of them are labeled as Uncertain. This suggests that part of the performance discrepancy arises from the distinct thresholds each method uses to classify a variant as benign. AM applies a general threshold designed to operate across the entire human proteome, regardless of protein region or functional context. In contrast, MotSASi uses a threshold specifically optimized for SLiMs in the context of their interactions with protein partners. The second key factor relates to the intrinsic nature of SLiMs and their interactions with their receptor domains, as reflected in the regular expressions (RE) that define them. Typically, some positions within a RE are structurally constrained and tolerate only a limited set of amino acids (“rigid positions”), whereas others are more permissive, allowing for greater sequence variability (“flexible positions”). We assessed the performance of MotSASi and AM separately on variants located in each type of position. While both algorithms performed similarly on flexible positions (MCC: MotSASi 0.50 vs. AM 0.37), the difference was considerably more pronounced for rigid positions (MCC: MotSASi 0.88 vs. AM 0.41). These results are consistent with the design of each method. AM is a general-purpose predictor that evaluates the variant in isolation, without explicitly modeling protein–protein interactions. By contrast, MotSASi directly assesses the variant within the structural context of the motif–domain complex, enabling it to better capture the functional consequences of mutations at key binding

residues. Since flexible positions typically do not make direct contacts with the receptor, both methods show comparable predictive performance for those variants."

iii) Concerning the third issue, we would like to highlight that the GO terms, as in the original MotSASi framework, primarily serve as a control for subcellular localization consistency. They ensure that the protein containing the potential motif resides in compartments compatible with those of the known interactors for that motif class as curated in the ELM database. For example, the epididymal secretory glutathione peroxidase (UniProt ID: O75715), encoded by the GPX5 gene, contains a match to the DOC_MAPK_JIP1_4 motif class at positions 90–96 (KPYGLVV). However, GPX5 is annotated with the GO cellular component term "extracellular region," which is not associated with any of the true positive ELM entries for this motif class. As a result, this candidate is discarded as a likely non-functional SLiM. Many similar examples were documented in our original MotSASi publication. We have included explicitly this type of analysis in the revised version of the manuscript, in the broader discussion mentioned in point i).

Point 2) The authors state "Our pipeline significantly expanded the repertoire of known functionally" but since these are predictions only and not functional assays, this must be corrected.

We thank the reviewer for this important observation. The corresponding statement has been revised in the manuscript to reflect that our findings are predictive in nature. We now explicitly acknowledge that functional experimental validation is required to confirm the physiological and pathophysiological relevance of the predicted SLiMs. Specifically, in the revised version of the manuscript, we added the following statement:

Lines [598-601]: "Our pipeline significantly expanded the repertoire of predicted functionally relevant SLiMs, achieving an almost 22-fold increase in the number of candidate motifs analyzed across the whole human proteome. These predictions remain to be validated by functional assays to confirm their physiological and pathophysiological relevance."
